# Transcriptional Dynamics Reveal Critical Roles for Non-coding RNAs in the Immediate-Early Response

**DOI:** 10.1371/journal.pcbi.1004217

**Published:** 2015-04-17

**Authors:** Stuart Aitken, Shigeyuki Magi, Ahmad M. N. Alhendi, Masayoshi Itoh, Hideya Kawaji, Timo Lassmann, Carsten O. Daub, Erik Arner, Piero Carninci, Alistair R. R. Forrest, Yoshihide Hayashizaki, the FANTOM Consortium, Levon M. Khachigian, Mariko Okada-Hatakeyama, Colin A. Semple

**Affiliations:** 1 MRC Institute of Genetics and Molecular Medicine, University of Edinburgh, Edinburgh, United Kingdom; 2 Laboratory for Integrated Cellular Systems, RIKEN Center for Integrative Medical Sciences, IMS, W518, Suehiro-cho, Tsurumi-ku, Yokohama, Japan; 3 UNSW Centre for Vascular Research, University of New South Wales, Sydney, New South Wales, Australia; 4 RIKEN Center for Life Science Technologies, Division of Genomic Technologies, Yokohama, Kanagawa, Japan; 5 RIKEN Preventive Medicine and Diagnosis Innovation Program, Wako, Saitama, Japan; 6 RIKEN Omics Science Center, Yokohama, Kanagawa, Japan; Ecole Normale Supérieure, FRANCE

## Abstract

The immediate-early response mediates cell fate in response to a variety of extracellular stimuli and is dysregulated in many cancers. However, the specificity of the response across stimuli and cell types, and the roles of non-coding RNAs are not well understood. Using a large collection of densely-sampled time series expression data we have examined the induction of the immediate-early response in unparalleled detail, across cell types and stimuli. We exploit cap analysis of gene expression (CAGE) time series datasets to directly measure promoter activities over time. Using a novel analysis method for time series data we identify transcripts with expression patterns that closely resemble the dynamics of known immediate-early genes (IEGs) and this enables a comprehensive comparative study of these genes and their chromatin state. Surprisingly, these data suggest that the earliest transcriptional responses often involve promoters generating non-coding RNAs, many of which are produced in advance of canonical protein-coding IEGs. IEGs are known to be capable of induction without de novo protein synthesis. Consistent with this, we find that the response of both protein-coding and non-coding RNA IEGs can be explained by their transcriptionally poised, permissive chromatin state prior to stimulation. We also explore the function of non-coding RNAs in the attenuation of the immediate early response in a small RNA sequencing dataset matched to the CAGE data: We identify a novel set of microRNAs responsible for the attenuation of the IEG response in an estrogen receptor positive cancer cell line. Our computational statistical method is well suited to meta-analyses as there is no requirement for transcripts to pass thresholds for significant differential expression between time points, and it is agnostic to the number of time points per dataset.

## Author Summary

Cells respond to stimuli through a set of genes that are primed for rapid activation. These genes, known as immediate-early genes (IEGs), are regulated at the level of transcription of the messenger RNA, and at subsequent RNA processing levels. These rapid responders are then rapidly switched off in normal cells. Immediate-early genes are involved in many cellular processes, including differentiation and proliferation, that are often dysregulated in cancer where they become continuously active. We characterise IEGs in a genome-wide sequencing dataset that captures their transcriptional response over time. Using a novel analysis technique, we identify both protein-coding and non-coding genes that are activated comparably to IEGs and investigate their properties. We examine how IEGs are switched off, including through microRNAs, small non-coding RNAs that act to control the level of key IEGs. We identify a novel set of microRNAs responsible for the attenuation of the IEG response in an estrogen receptor positive cancer cell line.

## Introduction

Immediate-early (or primary response) genes are induced in response to a stimulus without the requirement of de novo protein synthesis [1]. The source and duration of the induction signal can determine alternative cell fates, for example, transient signalling may result in cell proliferation, whereas sustained signalling gives rise to cell differentiation [2]. The activation of ErbB receptors by epidermal growth factor (EGF) or heregulin (HRG) in the MCF7 breast cancer cell line exemplifies the impact of such transient or sustained signalling on cell fate [3, 4]. The well-studied mitogen-activated kinase (MAPK), and in particular extracellular signal-regulated kinase (ERK) pathways, play important roles in signal transduction in the immediate-early response as well as many other cellular responses [1]. The over-expression of immediate early genes is correlated with cancer progression, and some of the best studied are known oncogenes [5]. However, in spite of the biomedical importance of the immediate-early response, our understanding of both its initiation and attenuation is far from complete. We lack a comprehensive account of how the mechanisms underlying these phenomena vary across stimuli and cell types, and few studies have explored the full diversity of transcripts involved

Many immediate-early genes (IEGs) encode transcription factors which regulate secondary response genes (SRGs) [6]. Necessarily, there is a delay in the expression of SRGs since, unlike IEGs, they require de novo protein synthesis. However a set of delayed IEGs may also be present concurrently with SRGs which can complicate efforts to study IEGs. It is believed that delayed IEGs can be identified by their increased length, greater number of exons and lack of transcription factor activity in addition to the delayed timing of their expression in comparison with typical IEGs [6]. Delayed IEGs also typically lack the conserved binding sites for SRF, NF-*κ*B and CREB generally found in IEGs [6]. Chromatin architecture plays a critical role in IEG expression [7]. The presence of CpG islands and constitutively active chromatin, high polymerase densities at promoters that may indicate a role for the regulation of RNAPII, and a single CAGE peak at the promoter are all features reported to be associated with IEGs [1, 6]. However, many studies have been restricted to a limited number of promoters, and examine a single cell type and stimulus. The diversity of cell types, stimuli and genes investigated in the literature makes it difficult to generalize about the molecular mechanisms underlying the induction of even the best studied IEGs [8].

A more comprehensive understanding of the initiation of the immediate-early response, and its less well studied attenuation are required. Studies of IEG induction show distinct differences between the kinetics of pre-mRNA and mature mRNA which are particularly evident for delayed IEGs where the mature mRNA may peak up to 3 hours later than the precursor [6]. A transient overshoot in pre-mRNA production has been proposed as a strategy to shape the timing and magnitude of response in the face of the slow mRNA degradation kinetics that would otherwise determine the kinetics [9]. The transient induction of phosphorylated c-Fos in MCF7 cells in response to HRG is thought to be due to multiple negative feedback loops in the signalling and transcription network [3, 4]. The attenuation of the initial response of delayed IEGs to EGF was also shown to depend on negative feedback through de novo transcription in HeLa cells [10], but it is unknown whether negative feedback plays roles in other cell types or under other stimuli.

IEG expression is tightly regulated at multiple levels, including control of transcription initiation and elongation as well as subsequent co-transcriptional and post-transcriptional events [1]. Recent studies have implicated splicing as an important factor controlling IEG expression, such that the FOS locus can remain transcriptionally active long after spliced mRNA production has ceased [11]. Others have established important roles for mature miRNAs in IEG regulation, with an early decrease in miRNA abundance permitting rapid induction of IEGs [12]. On the attenuation of the immediate-early response, detailed kinetic modelling of the transient upregulation of the Atf3 transcription factor (an inhibitor of Egr1) has concluded that, following induction, the mechanism whereby Atf3 is rapidly repressed is likely to involve newly-synthesised miRNA [13]. The transcription of primary miRNA transcripts (pri-miRNAs), and the subsequent role of the mature transcripts in the immediate-early response is unexplored in genome-wide data.

There has been intense interest in the roles of long non-coding RNAs (lncRNAs) in cellular differentiation. This class of transcripts is currently under-studied, but lncRNAs are differentially expressed during differentiation, are preferentially localised in chromatin and have been proposed to ‘fine-tune’ cell fate via their roles in transcriptional regulation [14–16]. Genome-wide characterisation of histone modifications H3K4me3 and H3K27me3 at lncRNA has demonstrated common features with mRNA, whereas patterns of DNA methylation differ [17]. Until now we have lacked a comprehensive study of non-coding RNA (ncRNA) species active in the immediate-early response, encompassing different cell types and stimuli.

The FANTOM5 project has recently produced the most comprehensive expression atlas for human and mouse cells, based upon cap analysis of gene expression (CAGE) data [18]. In particular, the CAGE time series datasets obtained for MCF-7 cells and human primary aortic smooth muscle cells are a unique resource for the study of the temporal response of stimulated human cells [19]. As CAGE data is obtained from the 5’ end of capped mRNA transcripts, it is expected to reflect the initial burst of overproduction of mRNA at promoters better than other expression data, and hence is well suited to explore the immediate-early response. Using these unique datasets, and a novel approach to time series analysis, we identify a comprehensive set of transcripts whose expression patterns are altered in response to a stimulus genome-wide, including all ncRNA transcripts present. We define kinetic signatures as response patterns corresponding to the likely solutions of kinetic models, including a signature representing the classical IEG response. Transcripts are categorised according to the kinetic signature they fit best, if any, and the categories are then explored to identify the over-representation of known IEGs and the known characteristics of IEGs. Our methods are well suited to meta-analyses, and we are able to rigorously compare transcript classifications in the immediate-early responses of different cell types under different extracellular stimuli, revealing novel commonalities among a diverse array of cell type and stimulus specific transcripts. This work is part of the FANTOM5 project. Data downloads, genomic tools and co-published manuscripts are summarised here http://fantom.gsc.riken.jp/5/.

## Results

**Fig 1:**
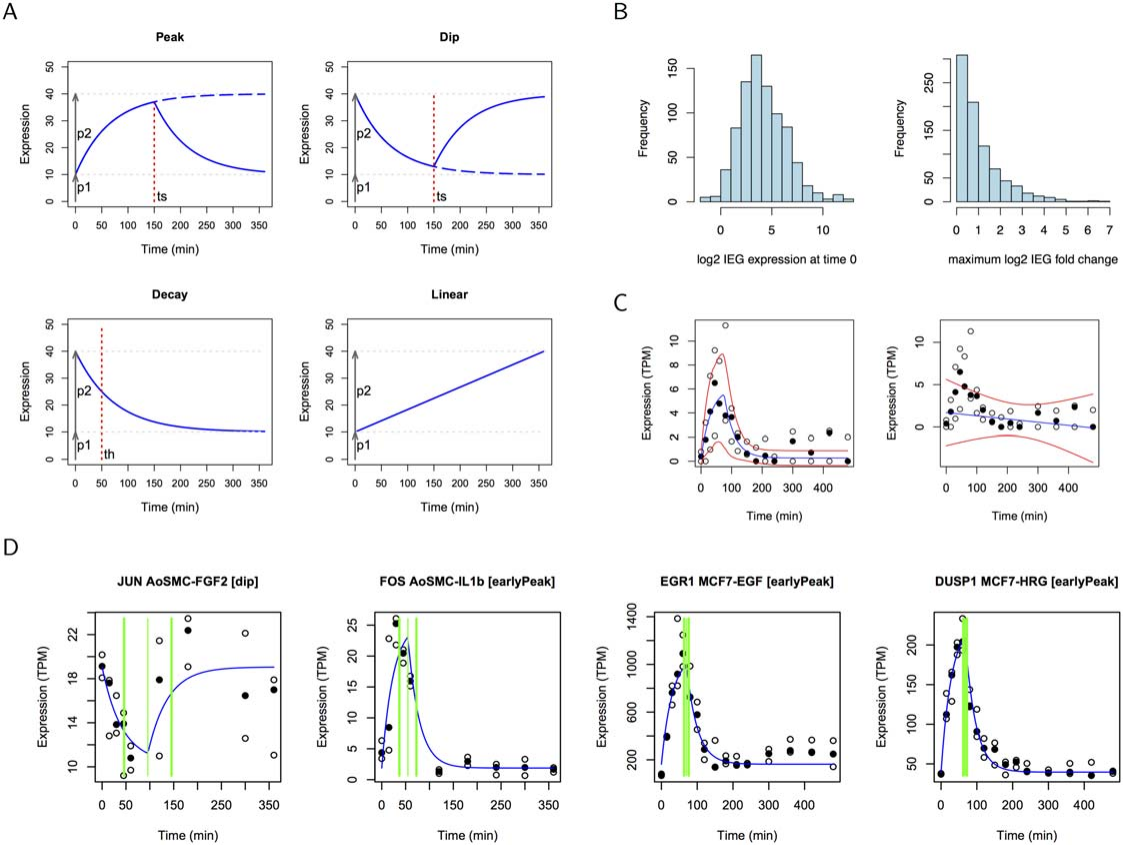
Kinetic signatures for IEGs. (A) Kinetic signatures are defined as piece-wise exponential (peak and dip), simple exponential (decay) or linear functions.(B) CAGE clusters associated with known IEGs show significant expression at time 0 (left; median 14.7 TPM). The maximum log2 fold change at any point in the time course over expression at time 0 is typically less than 2 (right; median 1.64). Histograms show data from all four data sets for 194l known IEGs. (C) Kinetic signatures fitted to the CAGE time course of EGR1 in EGF treated MCF7 cells yield values for the fit (log Z) and estimates for parameter moments. Plots show the kinetic signature function using computed parameter means (blue) and confidence intervals (red) for peak (left) and linear (right) kinetic signatures. In this case, log Z for the peak signature (-27.2) is greater than that for the linear model (-35), indicating a significantly better explanation of the data. Data values are plotted as circles (median value is filled). (D) CAGE time course data and best-fitting kinetic signature for IEGs JUN, FOS, EGR1 and DUSP1 (colours as in (C)). The vertical green lines indicate the mean switch time *t*_*S*_ and one standard deviation above and below.

Four kinetic signature functions were defined as illustrated in Fig 1A (see Materials and methods for details). These patterns were intended to capture mRNA transcription in response to a stimulus. Such exponential kinetics are characteristic of formalised systems biology models (comparable with observed and modelled mRNA and pre-mRNA expression in [4, 9]), and may reflect changes in both transcription and degradation rates over time [20]. The genome-wide CAGE data considered here necessarily included transcripts whose functions are unknown thus we began by hypothesising the possible kinetics they may display, rather than by constructing a detailed, interconnected systems model. Kinetic signatures serve as prototypical patterns reflecting changes in regulation, and are used here as a means to categorise time course responses for each transcript present. We focused on four particular time series datasets: human aortic smooth muscle cells (AoSMC) treated with FGF2 and with IL-1*β* (9 time points from 0 to 360 min; 3 replicates per treatment; IL-1*β* will be referred to as IL1b hereafter), as well as human MCF7 breast cancer cells treated with EGF and HRG (16 time points from 0 to 480 min; 3 replicates per treatment). Aortic smooth muscle cells are primary cells which are components of blood vessels. They are normally growth-quiescent in the normal adult vessels, but are activated by injury, or exposure to growth factors (including FGF2) and pro-inflammatory cytokines (including IL1b). These cues are sensed by these cells through changes in immediate-early gene expression, and can lead to increased proliferation and migration.

All transcripts for protein-coding genes were represented by conservatively thresholded FANTOM5 CAGE clusters (10 TPM; approximately 3 copies per cell [18]). More than one CAGE cluster could be assigned to each Ensembl gene, since clusters indicate transcription start sites (TSSs) and many genes possess multiple alternative promoters. A minimum of 500,000 mapped tags were required for a sample to be included in the analysis, and at least two biological replicates per time point. This led to exclusion of five libraries from the AoSMC-FGF2 time series, three from AoSMC-IL1b and one from MCF7-EGF. Supplementary text 1 of Arner *et al.* (2015) [19], and references therein, describes CAGE library preparation, CAGE library quality control, sequencing and transcription start site clustering. Supplementary text 2 of [19] describes the quality control and experimental protocols for the AoSMC and MCF7 experiments relevant to the present manuscript, presenting both CAGE and qRT-PCR data for specific genes.

Peak and dip signatures were defined as piece-wise exponential functions parameterised by the basal expression (*p_1_*), maximal change in expression (*p_2_*) and time of the change (*t_*s*_*). The peak and dip signature functions require a rate constant *δ* which is not an explicit parameter of the model. Instead, the rate is calculated from the switch time *t_*s*_* (see Eqs 1 and 2 in Materials and methods) and the piece-wise function specifies that the response reaches 90% of *p_2_* at *t_*s*_*. This formulation ensures that the initial rise in expression shows an exponential characteristic that is not limited to the almost linear initial change in expression that might otherwise result from a small value of the rate constant *δ*. See Materials and methods for additional details of model definitions.

CAGE clusters assigned to approximately 200 known IEGs showed significantly elevated expression at the start of the time course, hence the importance of including the *p*_1_ parameter in the kinetic signatures (see Fig 1B). The ratio of the maximum expression to expression at time 0 was typically less than 2 as shown in Fig 1B. For the analysis of time series in this context, we distinguished early peaks from late peaks by bounding the prior range for the *t_*s*_* parameter by 1-240 minutes (the first half of the time series), and by 240 minutes-end time (the second half) of the experiment respectively.

The fit between kinetic signatures and the time series CAGE data was assessed using the nested sampling algorithm to calculate the log of Bayesian evidence (also known as the marginal likelihood), log Z, using existing algorithms [21]. The likelihood function was derived on the basis of maximum entropy and is applicable to any time series dataset with replicated data. CAGE clusters were assigned to one of the exponential kinetic signatures or to the linear model according to the value of log Z. A cluster was assigned to a ‘no decision’ category if the values of log Z (and the associated standard deviations) computed for each model did not permit a clear assignment. An example of fitting early peak and linear models to an EGR1 time course is presented in Fig 1C. Further details of the specification of priors for model parameters, and model selection are given in Materials and methods.

### Core regulatory components in the immediate-early response

The percentage of CAGE time series that could be reliably annotated with a kinetic signature varied from 21%–39% across the cell types and protein coding or RNA biotypes. The assignment of clusters to signatures showed a slight preference for the early peak category (S1 Fig and S2 Fig). In contrast, relatively few clusters were assigned to the linear signature (due in part to the decision making procedure that aimed to distinguish non-linear from linear expression patterns). Time series that could not be confidently annotated were excluded from further analysis, see S3 Fig for examples where an annotation could and could not be made.

Assignments of time series to models allowed commonalities and differences between cell types and stimuli to be identified, even in the presence of varying numbers of time points sampled for each cell type. Eleven genes exhibited the early peak response in all four data sets, including eight known IEGs (FOS, FOSB, EGR1, DUSP1, CTGF, CYR61, SCRNP1, and FOSL1, see S4A Fig). Known IEGs JUN and TRIB1 were among the additional seven IEGs and five transcription factors that had this response in three of the four data sets, see S4 Fig for the numbers of Ensembl genes in the intersection of the kinetic signature categories across all data sets. The two MCF7 data sets had a greater number of common annotations than did the two AoSMC data sets. CAGE data and the fitted kinetic signatures for JUN, FOS, EGR1 and DUSP1 are plotted in Fig 1D (with additional examples in S5 Fig). Although immediate early genes are typically rapidly upregulated, they may also be downregulated as is the case for JUN in AoSMC-FGF2. Lists of the genes assigned to kinetic signatures and the corresponding model parameters are provided in Supporting File 1. The picture that emerges suggests commonalities at the core of the regulatory networks governing the immediate-early response in different scenarios, as seen in the known IEGs and TFs shared across cell types and stimuli. Beyond this are larger numbers of known and possibly novel IEGs, demonstrating the specificity of the response to each stimulus. In fact in each case the vast majority of genes assigned to the early peak signature (candidate IEGs or SRGs) were specific to a particular cell type and stimulus. This offers an explanation for the ambiguity in the literature on the immediate-early response, with remarkably diverse arrays of genes implicated across different studies [11].

### The early peak signature is enriched for IEGs and signalling pathways

Examining the transcript classifications more broadly we saw further commonalities shared among the responses to different stimuli. Considering CAGE clusters with kinetic signature classifications across datasets increased the recovery of known IEGs. The proportion of clusters associated with known IEGs increased from 1.8% in the data set as a whole to 3.0% in the set of clusters where a reliable classification to any signature could be made, and then to 5.1% in the set of early peak clusters. The early peak category was significantly over-represented for known IEGs as shown in Table 1. This set of 194 IEGs was curated from the literature by the FANTOM5 consortium (published as supplementary S6 Table in [19]). It should ne noted that this list may contain genes that are not necessarily activated rapidly in all cell types and by all stimuli. However, to qualify as an IEG these genes must be expressed without de novo protein synthesis. The enrichment reported in Table 1 is with respect to the set of clusters with any assigned kinetic signature, which is already enriched for IEGs (p < 0.002). The measure of effect size is the odds ratio (the proportion of clusters in a specific category that are IEGs, divided by the corresponding proportion of non-IEGs in that category).

**Table 1:** Enrichment of IEGs in kinetic signatures.

**Data set**	**Early peak**	**Late peak**	**Dip**	**Decay**	**Linear**
AoSMC-FGF2	**1.4 (p = 0.022)**	0.35 (p = 1)	0.64 (p = 0.98)	**1.9 (p = 3.2e-3)**	0 (p = 1)
AoSMC-IL1b	1.1 (p = 0.33)	**1.8 (p = 5.1e-05)**	0.25 (p = 1)	0.6 (p = 0.96)	0 (p = 1)
MCF7-EGF	**1.7 (p = 9.6e-07)**	0.43 (p = 1)	0.91 (p = 0.76)	0.23 (p = 1)	0 (p = 1)
MCF7-HRG	**2.6 (p < 1e-16)**	0.78 (p = 0.96)	0.31 (p = 1)	0.29 (p = 1)	0 (p = 1)
All data sets	**1.7 (p < 1e-16)**	0.89 (p = 0.91)	0.53 (p = 1)	0.6 (p = 1)	0 (p = 1)

Enrichment (odds ratio) and p values by data set, and for all data sets (significant enrichments are in bold: p ≤ 0.05 by hypergeometric test).

Notably, IEGs were enriched in the early peak category without specifying a particular threshold on *t*_*s*_. Our categorisation according to the shape of the response identified a larger set of genes that retained enrichment for known IEGs in comparison with an alternative approach where we looked for enrichment of IEGs within early peak genes within specific bounds on *t*_*s*_. As will be demonstrated below, IEGs have *t*_*s*_ values across the 1-240 min range hence an approach based on thresholds is not likely to succeed. Therefore the early peak category captures an expression pattern common to IEGs, and thereby enhances the detection of IEGs. Again, classifications of the data into distinct characteristic profiles appear most useful for large time series datasets.

Gene ontology terms associated with early peak clusters that were significantly over-represented in HRG treated MCF7 cells are listed in S1 Table, see also S6 Fig (analysis performed with GOrilla [22] and REVIGO [23]). The terms listed in S1 Table were also over-represented in the set of early peak genes when all four data sets were combined (but did not have a significant q value after multiple-testing correction in this case). Terms relevant to the immediate-early response included *regulation of gene expression*, *regulation of transcription from RNA polymerase II promoter*, *regulation of RNA metabolic process* and *regulation of metabolic process*. In addition, *RNA splicing* and several associated terms were found in all four data sets combined, but with q values exceeding 0.05 (p = 9.57E-05; q = 0.07), indicating that early peak genes have a major impact on the regulation of transcription and splicing, consistent with previous studies of IEGs [1]. When gene lists were combined in this way, the number of genes in the target set increased from around 10% to 30% of the background set hence enrichment was more difficult to demonstrate.

Pathways in the Panther database [24] that were over-represented in gene lists derived from annotated CAGE clusters in all four experiments are listed in Table 2. Pathways associated with the early peak signature included the transforming growth factor (TGF) beta signalling pathway, and the platelet derived growth factor (PDGF) signalling pathway, which play critical roles in cellular proliferation and development [24] and shares the downstream targets with the ErbB receptor signalling pathway, the receptors for EGF and HRG and all those belong to the members of receptor tyrosine kinases (RTK) family. RTK signalling is initiated upon binding of ligands to the corresponding receptor complex and this leads to the phosphorylation of other cellular proteins. The phosphatidylinositol 3 (PI3K) pathway is also over-represented. PI3K binds to tyrosine phosphopeptide sites of receptors serving diverse functions. RTKs can stimulate cells through either the MAPK pathway, the AKT/PI3K pathway, or a combination of the two [25]. The promoters of IEGs JUN and FOS contain a number of elements that are targets for MAPK signalling [26]. The TGF-beta, integrin and Toll-like receptor (stimulating NF-*κ*B) signalling pathways also provide a route for signals to pass from the extracellular environment to the nucleus [5, 27, 28]. Pathway analysis indicates that kinetic signatures identify the transcription of genes linked to the RTK, AKT, MAPK and NF-*κ*B pathways.

**Table 2:** Pathway analysis.

**Pathway**	**Signature**	**P value**
TGF-beta signaling pathway	early peak	2.2e-04*
Oxidative stress response	early peak	3.9e-04*
Platelet derived growth factor (PDGF) signaling pathway	early peak	5.2e-03
Angiotensin II-stimulated signaling through G proteins and beta-arrestin	early peak	0.024
Phosphatidylinositol 3 (PI3)-kinase pathway	early peak	0.016
Angiogenesis	early peak	0.021
Toll receptor signaling pathway	late peak	8.6e-05*
Integrin signalling pathway	late peak	8.5e-04*
Cadherin signaling pathway	late peak	8.4e-03
Apoptosis signaling pathway	late peak	0.037
Notch signaling pathway	decay	3.3e-03

P values for the over-representation of CAGE clusters in 73 Panther gene sets containing at least 20 genes. P values are calculated by hypergeometric test on the counts of clusters from all four data sets combined. Only pathways with p values ≤ 0.05 are listed (those with a FDR significant at 0.1 are indicated by *).

### Kinetics and chromatin features underlying IEG induction

Most CAGE clusters associated with known immediate early genes were assigned to the early peak signature where they constituted 5% of the total CAGE clusters exhibiting this behaviour. Values for *t*_*s*_ in the range 1-100 min were more prevalent for early peak clusters in comparison with clusters annotated with the dip signature (see S7 Fig). When the rates, *δ*, for early peak, dip and decay signatures were considered (note that the decay model is parameterised by the half life *t*_*h*_ rather than the switch time *t*_*s*_, see Eq 3 in Materials and methods), dip and decay signatures showed similar distributions to each other, and both were shifted towards higher values in comparison with early peak rates. This may indicate that the (pre) mRNA kinetics of switching-on are faster than switch-off kinetics as might be expected due to the latter being dominated by relatively slow mRNA degradation rates in many cases [9]. The distribution of *t*_*s*_ values for early peak clusters of known IEGs was consistent with the overall distribution (S7 Fig) which may indicate that the larger set of transcripts we assigned to the early peak signature may be part of a regulatory module under the same control as IEGs, or may include uncharacterised IEGs.

#### Attenuation of the immediate-early response

**Fig 2:**
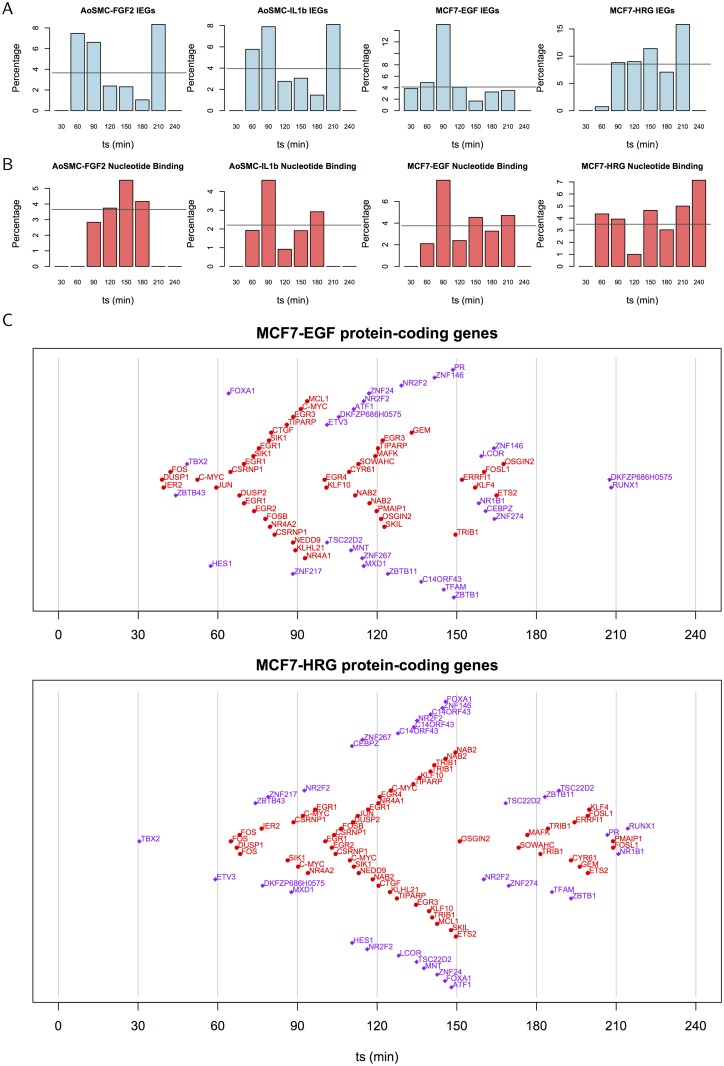
Timing of early peak CAGE clusters. (A) Bar charts showing the percentage of early peak clusters associated with IEGs (*t*_*s*_ binned in 30 min intervals), and (B) and those associated with nucleotide binding genes. The horizontal line indicates the average percentage. (C) The timing of known IEGs and transcription factors is shown for IEGs (red) and TFs (purple) assigned to the early peak signature in each MCF7 experiment. Symbols indicate the *t*_*s*_ (plotted on the x axis) and are labelled with the gene name associated with the CAGE cluster (symbols are positioned on the y axis for legibility only).

It has been proposed that nucleotide binding proteins are among the feedback regulators responsible for the attenuation of the immediate-early response to EGF [10]. This set of delayed early genes has been shown to be activated in waves following FOS, JUN and EGR1 expression in HeLa cells [10]. Following Amit *et al.* (2007) [10], we constructed a set of 444 genes assigned with the Gene Ontology annotation for nucleotide binding GO:0000166 (IEA assignments were excluded). Only three of these genes were transcription factors and six were IEGs therefore these sets were essentially disjoint. The set of nucleotide binding genes was over-represented in the early peak signature in all four data sets combined (p = 0.007), and for AoSMC-FGF2 and MCF7-EGF data sets individually (p = 0.018 and p = 0.003 respectively). The timing of immediate early and nucleotide binding gene expression is shown in Fig 2A and 2B where it can be seen that in AoSMC-FGF2, AoSMC-IL1b and MCF7-EGF data the largest proportion of known IEGs is found in the 30-90 min interval when *t*_*s*_ values are binned in 30 min intervals (the proportion of clusters annotated to known IEGs is expressed as a percentage of all clusters within each 30 min period according to *t*_*s*_). This pattern was less apparent in the MCF7-HRG cell line where the proportion of known IEGs found in an interval exceeded the overall average towards the end of the time course. Further, in AoSMC-FGF2, AoSMC-IL1b and HRG treated MCF7 cells there was a peak in IEGs around 3 hours after stimulus (180-210min) suggesting genes currently described as IEGs may also have a role later in the immediate-early response than would be expected. Surprisingly, the proportion of nucleotide binding genes was maximal in the 60-90 min interval for the AoSMC-IL1b and MCF7-EGF data, and in all cases many nucleotide binding genes were activated concurrently with IEGs in contrast with previous reports [10]. It is also worth noting that significant downregulation did not occur until the second hour, and this may require both early induction of transcriptional repressors and the RNA degradation proteins BTG2 and ZFP36 (tristetraprolin) [29].

Thus, cellular responses to FGF2, EGF, IL1b and HRG may be distinguished by the variable timing of factors (whether they are known IEGs or nucleotide binding proteins) that constitute and repress each response.

Further exploration of model parameters yielded other insights. The timing of IEG induction and that of known transcription factors (TFs) is contrasted in Fig 2C where a relatively consistent pattern of IEG activation beginning with FOS, DUSP1 and IER2, and continuing with JUN, C-MYC, EGR1 and DUSP2 can be seen. A number of non-IEG transcription factors were also activated: TBX2 activates early and RUNX1 late in the timelines. After HRG stimulation, genes typically peak later than after EGF stimulation: on average, the genes listed in Fig 2C take 26 min longer to reach their peak after HRG stimulation in comparison with EGF stimulation. This may be related to the fact that the HRG-induced repressor of FOS transcription requires new protein synthesis, whereas this is not required following EGR1 induction [4]. The average difference between IL1b and FGF2 stimulation in AoSMC was approximately 10 min for genes assigned to the early peak category. See S8 Fig for timelines for Aortic smooth muscle cells and for non-coding genes.

Targets of the MAPK cascade in Fig 2C included the transcription factors ETV3 and KLF4 [30] as well as IEGs JUN and FOS [26]. The transcriptional repressor NAB2 peaked relatively late in both MCF7 time courses. This is consistent with reports that NAB2 represses EGR1 and thereby attenuates the immediate-early response in HeLa cells stimulated with EGF [10]. Nucleotide binding genes found to peak within 240 min in both MCF7 time courses included the transcription factor RUNX1 and three IEGs: TRIB1, SIK1 and GEM. Significant upregulation of NAB2, TRIB1 and GEM was previously found in MCF7 cells following EGF and HRG treatment (see S1 Table in [3]) These genes may play a role in the attenuation of the immediate-early response in MCF7 cells. Notably, physical interactions between RUNX1 and JUN, FOS and MYC are listed in BioGRID [31], as are interactions between TRIB1 and MYC, and NAB2 and both EGR1 and EGR2. Interactions between JUN and both FOS and MYC are also listed.

#### IEG transcription peaks within 120 minutes

Immediate early genes are typically shorter in length than the genome-wide average [6]. The mean length of genes for which we have CAGE cluster data was 64Kb, more than twice the mean length of the subset of known IEGs for which we have data (24Kb). The mean length of genes annotated with the early peak signature was close to the genome average (67Kb), indicating no enrichment for shorter genes, and hence that this category contains a mixture of IEGs, co-regulated and delayed early genes and their downstream targets.

**Fig 3:**
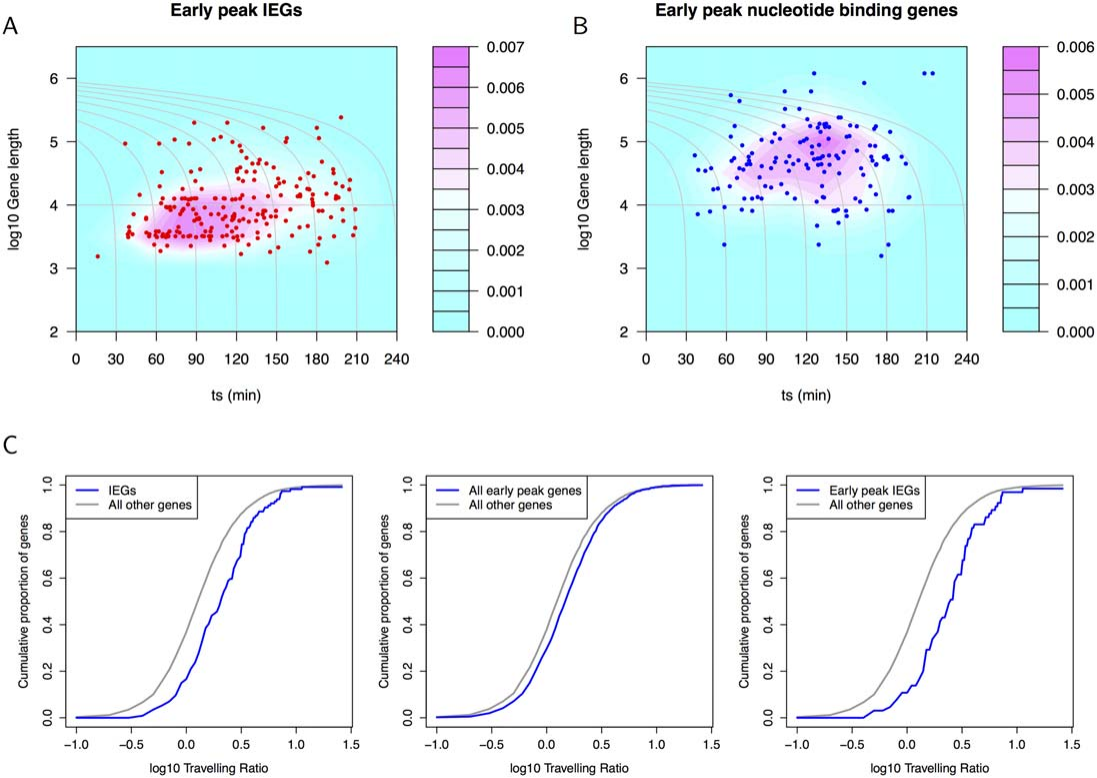
Density plots of gene length against *t*_*s*_ for early peak clusters. Grey contour lines indicate the projected *t*_*s*_ for the completion of transcription accounting for gene length (a transcription rate of 60 bases/s is assumed [32]). (A) Early peak known IEGs (red symbols represent the underlying IEG CAGE cluster data). (B) Early peak known nucleotide binding genes and underlying data (blue symbols). (C) Travelling ratios for known IEGs and for early peak genes in MCF7 cells demonstrate promoter proximal pausing as the travelling ratio is shifts towards higher values. The intersection of IEGs and early peak genes (right-most plot) shows that the strong pausing effect seen for IEGs holds for those assigned the early peak signature.

However, a weak positive correlation between gene length and *t*_*s*_ could be shown for early peak genes by Pearson correlation (all early peak genes: R = 0.10, p = 2.8e-11; known IEGs: R = 0.11, p = 1.2e-3; nucleotide binding genes: R = 0.11, p = 3.8e-5). Fig 3A and 3B contrast the density of gene length vs *t*_*s*_ for known IEGs assigned to the early peak signature with the densities of early peak nucleotide binding genes. Known IEGs were typically shorter in length and had lower *t*_*s*_ than nucleotide binding genes (combined data from all four datasets). Surprisingly, Fig 3A demonstrates that short IEGs ∼ 1-5Kb in length were activated with broad range of kinetics, from the lowest to the highest switch time *t*_*s*_. Thus the typically short length of IEGs will decrease the time required for their transcription, but IEGs are not necessarily induced with equally rapid kinetics. The time at which short IEGs reach their transcriptional peak was up to three hours after the stimulus suggesting their activation rates coordinate their expression with diverse processes and pathways: late-acting IEGs are not delayed due to gene length. Further, many early peak genes not known to be IEGs fell within the range of characteristics of known IEGs: length from 1.2Kb-240Kb, *t*_*s*_ less than 210 min.

The contour lines plotted in Fig 3A and 3B show the projected *t*_*s*_ for the completion of transcription given the estimate for the 5’ kinetics and the length of the gene. For example, the contour plotted at 120 min curves leftwards to identify those longer genes which were transcribed with lower *t*_*s*_ whose complete transcription would peak at 120 min (assuming equivalent splicing regulation). The density for known IEGs was predominantly within the 120 min contour. In contrast, the density for nucleotide binding proteins was beyond this contour. Comparing the counts of early peak IEGs, nucleotide binding genes and TFs within the 90 min contour with those outside this contour shows 28% of early peak IEGs, 17% of nucleotide binding genes and 22% of TFs lie within this contour in comparison with the 18% average for early peak genes. IEGs were significantly enriched within the 90 min contour (p = 5.1e-5 by hypergeometric test). Both early peak IEGs and TFs were enriched within the 120 min contour (p = 1.5e-4 and p = 0.04 respectively by hypergeometric test). While currently identified IEGs have distinct characteristics, our analysis also identifies a number of TFs induced on a similarly rapid timescale that may be part of the initial phase of the immediate-early response, or its attenuation.

#### Promoter-proximal pausing is prevalent in IEGs in MCF7 cells

The assignment of genes to kinetic signatures could also be used to explore their association with promoter-proximal pausing through calculation of the travelling ratio. The travelling ratios for the union of all genes with CAGE clusters annotated as early peak in the two MCF7 data sets and for the set of known IEGs are presented in Fig 3C. The travelling ratio is the ratio of RNAPII ChIP density at the promoter to that over the gene body [33]. We calculated the ratio of exon 1 density (read depth/locus covered) to the density over all other exons. Using the RNAPII ChIP data (control) from MCF7 cells published by Welboren *et al.*[34], we found IEGs to be associated with promoter-proximal pausing (that is, with a greater travelling ratio than non-IEGs; p = 1.2e-9 by Wilcoxon rank sum test on 114 IEGs compared with 8438 non-IEGs), and that the larger set of early peak genes was also associated with pausing (p = 1.4e-14; 1421 early peak genes compared with 7131 reference genes). The set of 65 early peak genes that were known to be IEGs shows notably high travelling ratios (p = 3.8e-10; 65 genes compared with 8487 reference genes). The travelling ratio plots in Fig 3C illustrate these significant shifts towards increased ratios between promotor-proximal (exon 1) and gene body RNAPII density as the cumulative density curves shift rightwards.

### Discovery of non-coding RNA genes active in the immediate-early response

CAGE clusters for RNA genes were assigned to kinetic signatures using the procedure described above. A lower threshold was used for the initial data selection: A minimum sum of 3 TPM normalised by relative log expression (RLE) over the time course was used as a threshold to increase the number of time courses from the more conservative 10 TPM criteria used for protein coding genes. S9 Fig shows the overlap between the assignments to clusters. LncRNA NEAT1 showed the early peak response in all four data sets, as did MALAT1 in three of the four sets. S10 Fig plots the CAGE data and kinetic signatures for NEAT1.

**Fig 4:**
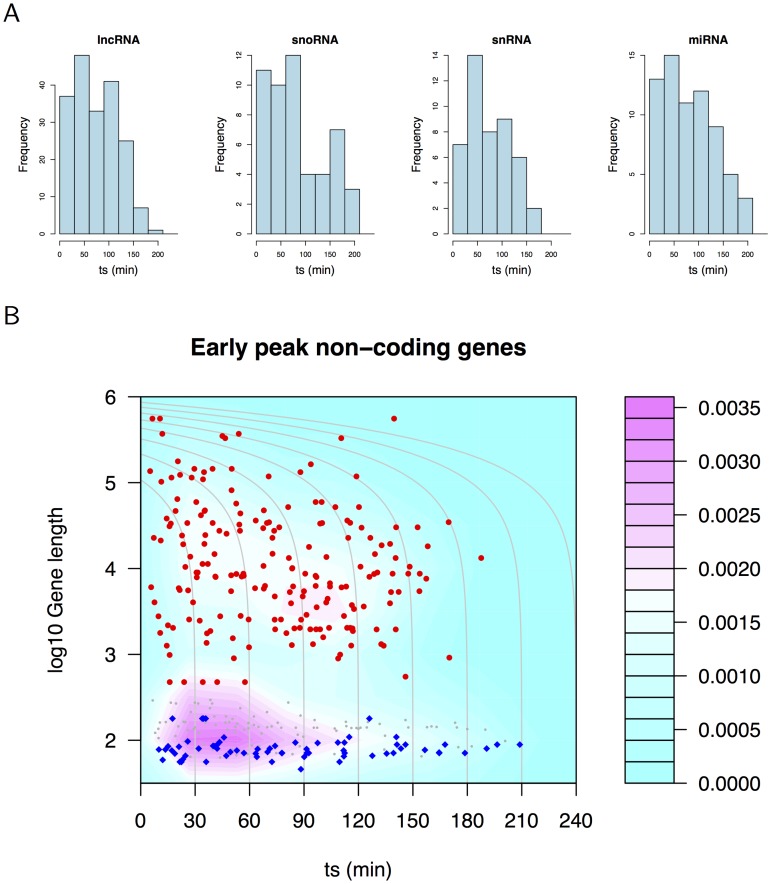
Non-coding RNA gene activation. (A) Histograms of *t*_*s*_ for lncRNA, snoRNA, snRNA and miRNA precursors show these genes are activated rapidly. (B) Density plot of early peak gene length against *t*_*s*_ for all RNA biotypes (grey symbols), lncRNA (red symbols) and miRNA precursors (blue symbols). LncRNA and miRNA form distinct clusters of RNAs activated with a wide range of kinetics.

The role of ncRNA in the immediate-early response is not well understood. A small number of mature miRNA that respond to EGF signalling have been identified [12, 35], and, in yeast, lncRNA have been shown to poise *GAL* genes for rapid activation [36]. Clusters assigned to the early peak signature were over-represented relative to other signatures for lncRNA and miRNA precursors (p = 0.013 and 3.8e-3, respectively, by hypergeometric test), and late peak and decay signatures were over-represented for snRNA (p = 1.9e-4 and 0.022 respectively). Thus lncRNA and miRNA precursors showed an analogous kinetic response to IEGs. The distributions of *t*_*s*_ for lncRNA, snoRNA, snRNA and miRNA assigned to the early peak category are shown in Fig 4A. These distributions can be compared with those for protein coding genes, including known IEGs (S7 Fig). These RNA biotypes had more rapid kinetics as shown by the number with *t*_*s*_ of less than 30 min.

The association between ncRNA *t*_*s*_ and length is shown in Fig 4B where it is apparent that lncRNA and miRNA precursors were activated with a range of kinetics—as was the case for known IEGs (compare with Fig 3). Naturally, these two classes of ncRNA have very different lengths, and miRNA precursors must be processed further to become part of an active complex.

#### Early peak CAGE clusters are located in open chromatin in MCF7 cells

Studies of known IEGs have suggested that they are transcribed from loci with constitutively permissive chromatin structure [1]. To determine the accessibility of CAGE clusters, read counts of DNaseI hypersensitivity data for MCF7 cells [37, 38] were determined for 200 bp windows centered at the midpoint of each protein-coding and non-coding MCF7 time course CAGE cluster. Protein-coding clusters assigned to the early peak category had significantly more reads than the remaining clusters (14% increase in mean read count, p = 2.1e-6 Wilcoxon rank sum test). Clusters associated with transcription factors were also in more accessible regions (p = 0.018), but, surprisingly, clusters assigned to known IEGs or nucleotide binding genes did not differ significantly from the reference set in either MCF7 time course (p > 0.08 by Wilcoxon rank sum test). We then explored the relationship between DNaseI counts and CAGE expression at time 0, maximum CAGE expression in the time course, and maximal fold change in CAGE expression across the time course for both MCF7 data sets. No predictive correlations were found between DNaseI and CAGE expression. However, we saw that CAGE expression at time 0 of greater than 10 TPM and DNaseI counts between 100 and 1000 were typical, whereas DNaseI counts of less than 100 were associated with TPM values of less than 10 at time 0. These observations suggest that a minimal level of chromatin accessibility is sufficient for the rapid activation of an immediate early gene on stimulus.

Considering the accessibility of CAGE clusters for non-coding genes, we found no significant difference between early peak clusters and the remaining set of non-coding clusters. However, early peak lncRNA had significantly greater DNaseI counts than the remaining non-coding clusters (40% increase in mean read count, p = 1.3e-8 Wilcoxon rank sum test). S11 Fig shows the distribution of counts for lncRNA resembles that of early peak protein-coding genes. Early peak miRNA precursors had fewer counts on average than the reference, but not significantly so.

Genome-wide analysis of enhancer activity was then performed. Multiple enhancer expression values arising from the many-to-many assignment of distal enhancers to genes [19, 39] were associated with genes by averaging. The mean enhancer expression of the union of early peak genes in MCF7 data was 74% of the mean in non early peak genes (2724 genes; p = 5.9e-07), and enhancer expression for IEGs was further reduced to 46% of the mean in non-IEGs (238 genes; p = 0.16). In contrast, enhancer activity for transcription factors was 30% greater than for non-TFs (901 genes; p = 2.5e-14).

These results indicate that the promoters of IEGs and early peak genes are in accessible chromatin (but not more so than average in the case of IEGs), and are poised for transcription as shown by the high travelling ratios, but prior to stimulus they are not driven by enhancer expression. It is likely that this state is determined by specific transcription factors for IEGs and early peak genes.

#### Non-coding RNA in the attenuation of the immediate-early response

It has been previously demonstrated that in response to EGF stimulation a set of 23 mature miRNA show a rapid reduction in expression that upregulates a large number of target mRNAs in non-tumorigenic MCF-10A breast epithelial cells [12]. These miRNA were named immediately downregulated miRNAs (ID-miRs) [12]. Many mRNA are repressed by more than one ID-miR, for example, EGR1 is targeted by hsa-mir-191 and hsa-mir-212. In contrast, mature hsa-mir-21 was previously found to be upregulated on EGF stimulation [12, 35, 40].

Observing that the transcription of the host gene for hsa-mir-155 (an ID-miR) showed clear peaks in the time courses of AoSMC-FGF2, AoSMC-IL1b and MCF7-HRG cells (S12 Fig), and that the precursor transcript of hsa-mir-21 showed early or late peaks in expression in three of the CAGE time course datasets we consider, we sought to investigate the relationship between miRNA-mediated repression and transcriptional attenuation, and to test whether or not kinetic signatures can be used to find correspondences between time course datasets.

Utilising a small RNA sequencing dataset for MCF7 cells stimulated with HRG (9 time points from 0 to 480 min; 3 replicates per treatment, minimum library size 5,340,873 reads), we applied the kinetic signature assignment procedure once again to identify regulated species. Of the 716 mirbase miRNA that passed a minimum expression criterion (sum of median normalised reads across the time course ≥ 10), 11.6% were assigned to a kinetic signature (earlyPeak: 25; latePeak: 6; decay: 33; dip: 18; linear: 1;) a somewhat lower proportion than for CAGE data reflecting a greater variation between replicates. S13 Fig shows examples of the assignment of mature miRNA time courses to the dip and decay kinetic signatures that we expected to observe. None of the miRNA assigned to dip or decay signatures are previously-described ID-miRs. S14 Fig presents plots of the time courses for the eleven ID-miRs in the dataset. Surprisingly, only three of the eleven ID-miRs are downregulated from time 0, others show peaked or increasing profiles (the variation in the data is such that none of the ID-miRs can be assigned to a kinetic signature with our usual statistical criteria). These data indicate that ID-miRs may play diverse regulatory roles that are dependent on cell type and stimulus.

**Fig 5:**
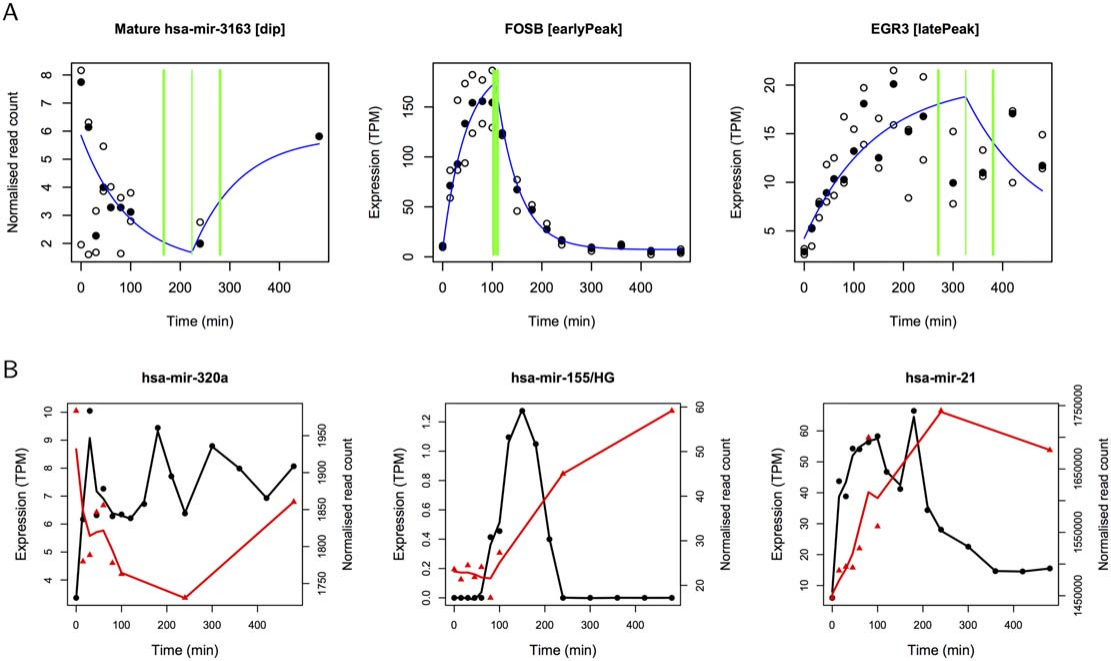
Mature microRNA regulation and host gene activation. (A) Expression of mature hsa-mir-6163 and transcriptional activation of two of its target IEGs FOSB and EGR3 in MCF7 cells in response to HRG. Data values are plotted as circles (median value is filled). (B) Median CAGE expression (black circles) of precursor miRNA and median mature miRNA expression (red triangles) for hsa-mir-320a, host lncRNA MIR155HG and mature hsa-mir-155, and for hsa-mir-21 in MCF7-HRG (three replicates, lines are a spline fitted to the data). For hsa-mir-320a the increase in CAGE expression is significant when comparing 0min and 210min and the decrease in mature transcript levels is significant when comparing 0min and 240min (p ≤ 0.05 by t test). For hsa-mir-155 the increase in CAGE expression of MIR155HG is significant when comparing 0min and 180min, and the increase in mature transcript levels is significant when comparing 0min and 240min (p ≤ 0.05 by t test). For hsa-mir-21 the increases in CAGE expression and in mature transcript levels are significant when comparing 0min and 80min (p ≤ 0.05 by t test).

Reasoning that miRNA-mediated repression will be reflected in the CAGE signals, either by direct action on mRNA or indirectly through transcriptional inactivation processes, we sought to establish a connection between the targets of mature miRNA that are assigned to the dip signature, and protein-coding genes with CAGE clusters assigned to the early peak signature in MCF7 cells stimulated with HRG. Making use of the TargetScan database of miRNA targets (version 6.2; http://www.targetscan.org) we found all targets for dip miRNAs. For 12 of the 15 dip miRNA present in TargetScan we observed a greater representation of miRNA targets in early peak genes than in a reference set of unregulated genes (namely, all protein-coding genes that could not be assigned to a kinetic signature in MCF7-HRG). The targets of seven of these miRNA were significantly overrepresented (by hypergeometric test): hsa-mir-139 (p = 2.6e-2); hsa-mir-224 (p = 1.1e-2); hsa-mir-522 (p = 1.5e-6); hsa-mir-548n (p = 9.8e-7), hsa-mir-676 (p = 3.2e-2), hsa-mir-3163 (p = 2.1e-7) and hsa-mir-3662 (p = 3.5e-3) and are candidate ID-miRs for MCF7 cells. The data for mature hsa-mir-3163 and two of its targets FOSB and EGR3 are shown in Fig 5A.

Of the four established ID-miRs for which we have CAGE data for the precursor, only two (hsa-mir-320a and hsa-mir-155) satisfied the expression criterion in the small RNA sequencing data. CAGE and miRNA expression data for these transcripts are presented together in Fig 5B where a lag between the rise in the CAGE signal and the recovery in the mature miRNA level for hsa-mir-320a can be seen, whereas the CAGE peak appears concurrent with the rise in mature hsa-mir-155 (none of these profiles satisfied our statistical criteria but significant changes occur between selected time points). Consistent with earlier reports on EGF stimulus, mature hsa-mir-21 increases in response to HRG as does the primary transcript (which was assigned to the early peak signature), see Fig 5B. Complex regulatory steps intervene between these two stages of miRNA maturity and impact on the stability of these transcripts hence there is no simple relationship between them. Further, it can be observed in Fig 5 and S13 Fig that there are often rapid fluctuations in mature miRNA in the first 100 min. Our kinetic models fit to the general trend which tends to have a minimum around 240 min, however, the rapid fluctuations may also be biologically significant in the first minutes after stimulus. S15 Fig plots the time course data for 27 mature miRNAs and the 41 CAGE clusters associated with their precursors in the MCF7-HRG experiment. Increases in CAGE expression may occur prior to increases in mature miRNA levels as can be seen for hsa-mir-21 and hsa-mir-222, or reductions in mature miRNA may occur in advance of reductions in CAGE expression as for hsa-mir-4800. It is readily apparent that there is no simple correlation between precursor and mature transcript abundance. A complete explanation of their relationship would account for the synthesis and stability of each species and is beyond the scope of the present manuscript.

It has long been known that miRNA host genes may be protein coding or lncRNAs [41]. As noted above, the host gene for hsa-mir-155 is a lncRNA that is assigned to the early peak signature in three CAGE datasets. We also found MIR99AHG (host gene for hsa-mir-99a, hsa-let-7c and hsa-mir-125b2) and two other lncRNA whose locus contains miRNA to be similarly transiently upregulated in two or more datasets. The miRNA in host gene MIR99AHG are predicted to target 11 IEGs, hsa-mir-155 targets 26, comparable with established ID-miRs hsa-mir-191 and hsa-mir-212 which target 4 and 8 respectively (these miRNA are not located within a lncRNA locus). Across the four CAGE datasets 22 distinct miRNA are within a lncRNA locus assigned to a transient kinetic signature, thereby collectively targeting 129 IEGs (73.3% of known IEGs present in the TargetScan database) indicating a considerable regulatory potential.

We have identified seven immediately and transiently downregulated mature miRNA that preferentially target early peak genes. While these regulatory miRNA differ from those previously associated with the immediate-early response, the regulatory role appears to be the same. The fine-tuning of signalling pathways by miRNA has been previously described [12, 13, 42–44] and our analysis demonstrates that this phenomenon can be identified in high-resolution time course data. Our findings indicate that a hypothesised negative feedback mechanism for Atf3-Egr1 kinetics that involves the synthesis of miRNA [13] may also be a more general feature of the immediate-early response. The relationship between precursor and mature miRNA transcripts is complex, however, our data suggests that downregulation of mature hsa-mir-320a is concurrent with increased transcription of the precursor transcript which can be expected to restore the repression of its target mRNAs which include DUSP4 and FOSL1 at a later phase of the immediate-early response. Transcriptional activation and repression of miRNA precursors in the immediate-early response is readily apparent in the small intersection of the datasets for MCF7-HRG.

## Discussion

We have defined kinetic signatures, including a signature representing the classical IEG response, as the basis for a novel approach to time series data analysis. We have shown that large numbers of transcripts can be categorised according to the kinetic signature their expression profiles fit best, if any, and the categories can then be explored using standard enrichment statistics. These methods have successfully identified known IEGs as well as many other transcripts displaying the known characteristics of IEGs. A Bayesian approach utilising the nested sampling algorithm [21, 45] is used to compare the fit of the models to the CAGE time series data, and in comparison with other methods, we find that more time courses can be assigned to kinetic signatures and with greater confidence. Model parameters also give the timing of potentially important events such as transitions in expression levels within the time course. Known IEGs are significantly enriched in CAGE clusters assigned to particular signatures (those involving early but transient upregulation within 240 minutes which we term the early peak response) and show other biological features of interest. In addition many relatively lowly expressed transcripts show expression profiles of interest, implicating the involvement of particular miRNA and lncRNAs in the immediate-early response.

Genes assigned to the early peak kinetic signature were over-represented in gene annotations and pathways that are biologically relevant to the immediate-early response, including *regulation of transcription from RNA polymerase II promoter* and the *TGF-beta signalling pathway*. Nucleotide binding genes have been proposed as components of the negative feedback architecture of the immediate-early response. We found these genes to be activated concurrently with IEGs in many cases, but, considering the time for completion of transcription where gene length is accounted for, the translation of these genes would peak later.

In common with known immediate-early genes, early peak genes showed promoter-proximal pausing in MCF7 cells, an RNAPII regulatory pathway previously demonstrated for selected IEGs in macrophages [7]. From consideration of DNaseI data, CAGE clusters for early peak genes and transcription factors tended to be located in accessible chromatin in MCF7 cells. The absence of a correlation between DNaseI counts and CAGE expression suggests that IEG promoters need not be located in the most accessible chromatin, rather a minimum level of accessibility is required and is not otherwise predictive of transcriptional activity. These results suggest that IEGs and early peak genes are primed prior to stimulus, with a permissive chromatin state maintained by transcription [7].

Intriguingly, certain lncRNAs had properties analogous to known IEGs. Such similarities and differences between the epigenetic regulation of lncRNA and mRNA have been reported previously in genome-wide data [17]. Many of these genes were activated rapidly after stimulus, and those assigned to the early peak signature originated from CAGE clusters in open chromatin. Further, regulated lncRNA are host to many miRNA targeting IEGs.

Mature miRNAs previously found to be immediately downregulated in response to EGF [12] were found to have diverse responses in the MCF7-HRG dataset. However, the mRNA targets of a number of immediately downregulated mature miRNA in this data were found preferentially in early peak (transiently upregulated) protein-coding genes in the independent, but matched CAGE dataset thus supporting the use of kinetic signatures to detect meaningful temporal patterns (with respect to known miRNA targets).

We identify hsa-mir-139 as an immediately downregulated miRNA: miR-139 is a known regulator of several canonical pathways in the metastatic cascade in MCF7 cells [46], and is predicted to target the TGF-beta and PI3 signalling cascades [46] that we identify here as targets of immediate early genes. Differences in the miRNAs that respond to stimulus in MCF7 cells in comparison with MCF10A cells [12] may be due to the former being estrogen receptor positive, and to the different receptors in the ErbB receptor family that are stimulated. The MCF7-HRG data analysed here is the response to a ErbB3/4 ligand whereas the ID-miRs identified by Avraham *et al*[12] are responding to a ErbB1/EGFR ligand. Such differences have implications for the translational potential of miRNAs in cancer [47].

LncRNA are typically expressed at very low levels (NEAT1 and MALAT1 being notable exceptions), as are precursor miRNA, making their analysis problematic for methods that require more strictly thresholded expression data. The model definition and selection methodology we present is not limited by expression level, for example by tests for differential expression, nor do we rely upon the arbitrary thresholding that is common in clustering analyses. Models are specified in advance, and selection is based on the integration of model parameters rather than from a point estimate of best values, an approach which can be sensitive to the optimisation procedure used. Gene sets assigned to the best fitting model can be tested for over-representation of established gene and pathway annotations, and can be integrated with genome-wide data sets to test additional hypotheses.

## Materials and Methods

### Definition of kinetic signatures

Peak and dip signatures are piece-wise exponential functions parameterised by the basal expression (*p_1_*), maximal change in expression (*p_2_*) and time of the change (*t_*s*_*). The decay signature is parameterised by the basal expression (*p_1_*), the maximal change in expression (*p_2_*, being the difference between the expression at time 0 and *p_1_*) and the half-life (*t_*h*_*). The linear model is parameterised by the expression at time 0 (*p_1_*) and the change in expression (*p_2_*) from which the rate of increase or decrease can be calculated. See Fig 1A for plots of these functions.

The peak and dip signature functions require a rate constant *δ* which is not an explicit parameter of the model. Instead, the rate is calculated from the switch time *t_*s*_* and the piece-wise function specifies that the response reaches 90% of the expression change *p_2_* at *t_*s*_*, see Eqs 1 and 2. This formulation ensures that the initial rise or fall in expression shows an exponential characteristic that is not limited to the almost linear characteristic that might otherwise result from a small value of the rate constant *δ*.

In the peak model, expression increases to 90% of the change in expression parameter *p_2_* at *t_*s*_* from the basal level *p_1_* (Eq 1). *δ* is defined in terms of *t_*s*_*.

$$\begin{array}{cccc} \delta & = & log\left(0.1\right)/t_{s} & \\ y & = & p_{1}+p_{2}\left(1-e^{\delta t}\right) & t\leq t_{s}\\ y & = & p_{1}+0.9p_{2}-0.9p_{2}\left(1-e^{\delta (t-t_{s})}\right) & t>t_{s} \end{array}$$ (1)

In the dip model, expression drops by 90% of the change in expression *p_2_* at *t_*s*_* from the initial level *p_1_*+*p_2_* (Eq 2; *δ* is again defined in terms of *t_*s*_*).

$$\begin{array}{cccc} \delta & = & log\left(0.1\right)/t_{s} & \\ y & = & p_{1}+p_{2}e^{\delta t} & t\leq t_{s}\\ y & = & p_{1}+0.1p_{2}+0.9p_{2}\left(1-e^{\delta (t-t_{s})}\right) & t>t_{s} \end{array}$$ (2)

In the decay model, expression starts at *p_1_*+*p_2_* and drops exponentially at rate *δ* towards *p_1_*. In this case *δ* is calculated from the half-life *t_*h*_* which is an explicit parameter of the decay model (Eq 3).

$$\begin{array}{cccc} \delta & = & log\left(2\right)/t_{h} & \\ y & = & p_{1}+p_{2}e^{-\delta t} & \end{array}$$ (3)

The fit between kinetic signatures and the time series CAGE data is assessed using the nested sampling algorithm to calculate the log of Bayesian evidence (also known as the marginal likelihood), log Z [21] from the likelihood function and the prior. All priors are selected uniformly from a range bounded by maximum and minimum values derived from the time course. A likelihood based on the l1-norm is defined by Eqs 4 and 5 [48]. Eq 4 defines the normalising constant *ε*_*t*_ as the expected value of the moduli of the difference between the replicate observations at time *t* (*x*_*t*_) and the value predicted by the kinetic model (*μ*_*t*_). The product of the probabilities of the median observation at time *t* (${\tilde{x}}_{t}$) defines the likelihood function for a time series *x* of *m* samples (Eq 5). Maximisation of this likelihood minimises the sum of the moduli of the residuals (rather than their squares) on the basis that the testable information is restricted to the expected value of the modulus of the difference between theory and experiment. Should we know both the mean and variance, maximum entropy considerations would lead instead to the Gaussian distribution [48]. The inference of model parameters from CAGE data for the early peak and linear models using nested sampling and the l1-based likelihood is illustrated in Fig 1C. Time points where the replicates are most dissimilar contribute least to the likelihood as *ε*_*t*_ is larger—as is desirable.

$$\begin{array}{c} \epsilon_{t}=\left\langle \right|x_{t}-\mu_{t}\left|\rangle =\int \right|x_{t}-\mu_{t}|p\left(x\right)d^{N}x \end{array}$$ (4)

$$\begin{array}{c} p\left(x|\left\{\mu_{t},\epsilon_{t}\right\}\right)=\prod_{t=1}^{m}\frac{1}{2\epsilon_{t}}\exp(-\frac{|{\tilde{x}}_{t}-\mu_{t}|}{\epsilon_{t}}) \end{array}$$ (5)

Bayesian evidence values and model parameter estimates (and their standard deviations) are computed using nested sampling for all signatures for each time series. CAGE clusters are assigned to one of the exponential kinetic signatures if log Z for that signature is greater than 10 times log Z for the linear model and log Z minus its standard deviation (sd) is greater than log Z plus the estimated sd for any other exponential signature (nested sampling computes log Z for parameters mapped to 0..1 and we used the resulting log Z for the unit cube for model comparison). Clusters are assigned to the linear signature if log Z is 10 times greater than log Z for all exponential signatures. This decision making procedure is designed to minimise the incorrect assignment of exponential signatures to essentially linear data, and has been validated using synthetic data. The theoretical basis of nested sampling is summarised in S1 Text.

## Supporting Information

S1 Text Methods. (PDF)

S1 Fig Assignment of CAGE clusters for protein-coding genes to kinetic signatures. (PDF)

S2 Fig Assignment of CAGE clusters for RNA genes to kinetic signatures. (PDF)

S3 Fig Examples of parameter inference by nested sampling for CAGE data from MCF7-HRG clusters. CAGE TPM values are plotted as circles (median value is filled), predictions of the kinetic signature models using parameter means are shown in blue and the vertical green lines indicate the mean *t*_*S*_ (or *t*_*h*_ in the case of the decay signature) and one standard deviation above and below.
(PDF)

S4 Fig Venn diagrams showing the overlaps between Ensembl Ids for protein-coding clusters assigned to kinetic signatures. (A) early peak; (B) late peak; (C) dip; (D) decay.
(PDF)

S5 Fig CAGE time course data and kinetic signatures for clusters associated with IEGs JUN, FOS, EGR1 and DUSP1 for all four data sets. CAGE TPM values are plotted as circles (median value is filled), predictions of the kinetic signature models using parameter means are shown in blue and the vertical green lines indicate the mean *t*_*S*_ and one standard deviation above and below.
(PDF)

S6 Fig GO term enrichment and clustering for genes assigned to the early peak signature in MCF7 cells treated with HRG. This analysis was performed with GOrilla [22] and REVIGO [23].
(PDF)

S7 Fig Histograms of *t*_*s*_ and half-lives in the exponential kinetic signatures. The distributions of *t*_*s*_ for early peak, late peak, dip signatures and the half-life of the decay signature are shown in blue, the distributions of the subsets of known IEGs in each category are shown in red. The apparent bimodal distibution of *t*_*s*_ for peak models is an artefact of the different choices of prior range. When a peak model is run without the early or late restriction the distribution of switch times is not bimodal, however, fewer time courses are assigned to the peak category.
(PDF)

S8 Fig The timing of known IEGs and transcription factors and non-coding genes assigned to the early peak signature. (A) The timing of known IEGs and transcription factors is shown for IEGs (red) and TFs (blue) assigned to the early peak signature in each AoSMC data set. (B) The timing of non-coding genes assigned to the early peak category is shown for lncRNA (red) and all other ncRNA (blue) in MCF7 data and (C) in AoSMC data. Symbols indicate the *t*_*s*_ (x axis) and are labelled with the gene name associated with the CAGE cluster.
(PDF)

S9 Fig Venn diagrams showing the overlaps between Ensembl Ids for non-coding CAGE clusters. (A) early peak; (B) late peak; (C) dip; (D) decay.
(PDF)

S10 Fig CAGE data and kinetic signatures for clusters associated with NEAT1 in all four data sets. CAGE TPM values are plotted as circles (median value is filled), predictions of the kinetic signature models using parameter means are shown in blue and the vertical green lines indicate the mean *t*_*S*_ and one standard deviation above and below.
(PDF)

S11 Fig Distribution of DNaseI counts for 200bp windows centered on CAGE clusters. (Top) Distributions of early peak DNaseI counts (blue) and non-early peak counts (black) for protein-coding CAGE clusters, and QQ plot. Early peak clusters have significantly higher counts. (Middle) Distributions of early peak DNaseI counts (blue) and non-early peak counts (black) for non-coding CAGE clusters, and QQ plot. There is no significant difference between the distributions. (Bottom) Distributions DNaseI counts for early peak lncRNA (blue) and all other counts (black) for non-coding CAGE clusters, and QQ plot. Early peak lncRNA clusters have significantly higher counts.
(PDF)

S12 Fig CAGE data and early peak model for clusters associated with the host lncRNA for hsa-mir-155. Data is presented for the host lncRNA of hsa-mir-155 (MIR155HG ENSG00000234883) in AoSMC-FGF2, AoSMC-Il1b and MCF7-HRG data sets (data for MCF7-EGF does not pass quality controls). CAGE TPM values are plotted as circles (median value is filled), predictions of the kinetic signature models using parameter means are shown in blue and the vertical green lines indicate the mean *t*_*S*_ and one standard deviation above and below.
(PDF)

S13 Fig Mature miRNA expression in MCF7 cells in response to HRG in the small RNA sequencing data. The data and kinetic signatures are presented for two mature miRNA assigned to the decay signature and for two miRNA assigned to the dip signature. Expression values are plotted as circles (median value is filled), predictions of the model using parameter means are shown in blue and the vertical green lines indicate the mean *t*_*h*_ (or *t*_*S*_) and one standard deviation above and below.
(PDF)

S14 Fig Mature ID-miR expression in MCF7 cells in response to HRG. Eleven ID-miRs were present in the small RNA sequencing data with expression above the minimum threshold. Expression values are plotted as circles (median value is filled), and the dashed purple lines indicate the best-fitting kinetic signature. None of the ID-miR assignments passed the standard statistical criteria, hence these assignments are not significant (NS) and plotted for information only. It is apparent that hsa-mir-155 increases linearly, and that hsa-mir-191 peaks early in the time course. A number of ID-miRs show the expected immediate downregulation including hsa-mir-212 and hsa-mir-320a.
(PDF)

S15 Fig CAGE expression of precursor miRNA and mature miRNA expression. Median CAGE expression (black circles) of precursor miRNA and median mature miRNA expression (red triangles). Lines are are a spline fitted to the data.
(PDF)

S1 Table Gene Ontology process term enrichment in MCF7-HRG cells. (PDF)

S1 File Archive of tab-delimited files containing the kinetic signature assignments to CAGE clusters and associated model parameters for all time courses. (GZ)
