# Correction: Transcriptional Dynamics Reveal Critical Roles for Non-coding RNAs in the Immediate-Early Response

**DOI:** 10.1371/journal.pcbi.1005279

**Published:** 2017-02-10

**Authors:** The PLOS Computational Biology Staff

Part of the affiliation for the authors Stuart Aitken and Colin A. Semple is missing. The corrected affiliation is as follows:

^1^ MRC Human Genetics Unit, Institute of Genetics and Molecular Medicine, University of Edinburgh, Edinburgh, United Kingdom

## References

[1] Aitken S, Magi S, Alhendi AMN, Itoh M, Kawaji H, Lassmann T, et al (2015) Transcriptional Dynamics Reveal Critical Roles for Non-coding RNAs in the Immediate-Early Response. PLoS Comput Biol 11(4): e1004217 doi: 10.1371/journal.pcbi.1004217 25885578 10.1371/journal.pcbi.1004217 PMC4401570

