# Supplementary material for: Transcriptional Dynamics Reveal Critical Roles for Non-coding RNAs in the Immediate-Early Response
Source: PLoS Comput Biol. 2015 Apr 17;11(4):e1004217. doi: 10.1371/journal.pcbi.1004217 (PMC4401570; doi:10.1371/journal.pcbi.1004217)
Supplement: S1 Text — (PDF) [file pcbi.1004217.s001.pdf]

Transcriptional dynamics reveal critical roles for non-coding RNAs in the immediate early response – Supplementary Text

Stuart Aitken[1,*], Shigeyuki Magi[2], Ahmad M.N. Alhendi[3], Masayoshi Itoh[4], Hideya Kawaji[4], Timo Lassmann[4], Carsten O. Daub[4], Erik Arner[4], Piero Carninci[4], Alistair R.R. Forrest[4], Yoshihide Hayashizaki[4], the FANTOM Consortium, Levon M. Khachigian[3], Mariko Okada-Hatakeyama[2] and Colin A. Semple[1]

January 28, 2015

1. MRC Institute of Genetics and Molecular Medicine, University of Edinburgh, Edinburgh, EH4 2XU, United Kingdom.
2. Laboratory for Integrated Cellular Systems, RIKEN Center for Integrative Medical Sciences, IMS, W518, 1-7-22 Suehiro-cho, Tsurumi-ku, Yokohama, 230-0045, Japan.
3. UNSW Centre for Vascular Research, University of New South Wales, Sydney NSW 2052, Australia.
4. RIKEN Centre for Life Science Technologies (CLST), 1-7-22 Suehiro-cho, Tsurumi-ku, Yokohama 230-0045, Japan.

* Corresponding Author: Stuart.Aitken@igmm.ed.ac.uk

Methods

Samples and treatment

MCF7 human breast cancer cell line was provided to the FANTOM consortium for sequencing by Mariko Okada-Hatakeyama (Laboratory for Integrated Cellular Systems, RIKEN Center for Integrated Medical Sciences, IMS, Yokohama, Japan). MCF7 cells were obtained from American Type Culture Center (ATCC) and maintained DMEM supplemented with 10% fatal bovine serum.

Human AoSMCs were provided to the FANTOM consortium for sequencing by Levon Khachigian (UNSW Centre for Vascular Research, University of New South Wales, Sydney, Australia). Human AoSMCs were obtained from Cell Applications (CA, USA) and grown in Waymouth's medium, pH 7.4, supplemented with 1 mM L-glutamine, 10 units/ml penicillin, 10 mcg/ml streptomycin and 10% fetal bovine serum. The cells were seeded into 10 cm plates and at 80-90% confluency, washed with PBS and incubated in serum free medium for 24 hours. The cells were then incubated in serum free medium containing FGF-2 (50ng/ml) or IL-1beta (10ng/ml) for 15, 30, 45, 60, 120, 180, 240, 300 or 360 min. 0 min samples represent material harvested from serum starved and unstimulated cells. Total RNA was then harvested using Trizol reagent method. The RNA was quantitated on a Nanodrop spectrophotometer and sent to RIKEN Yokohama Institute for CAGE analysis after the samples were validated for inducible EGR-1 expression (Arner *et al.* 2015). Preparation of CAGE samples and quality control data is further described in (Arner *et al.* 2015).

Nested sampling

Nested sampling calculates the central results of Bayesian inference: the posterior distribution $P(\theta|D, H_i)$ of the parameters θ, and the evidence $P(D|H_i)$, that is, the support for the data D under hypothesis H_i

(Skilling 2006). The following summary is extracted from (Aitken and Akman 2013). Two models H_0 and H_1 can be compared through the ratio of their posterior probabilities (1), a calculation that can be decomposed into the Bayesian evidence (Z) and the prior probability of the respective hypotheses which may favour one model over another. The evidence (2) is a scalar quantity that can be viewed as an integral over the elements of mass ($\mathrm{d}X$) associated with the prior density $\pi(\theta)$.

$$\frac{P(H_1|D)}{P(H_0|D)} = \frac{P(D|H_1)P(H_1)}{P(D|H_0)P(H_0)} = \frac{Z_1 P(H_1)}{Z_0 P(H_0)} \tag{1}$$

$$Z = \int L(\theta)\pi(\theta)\,\mathrm{d}\theta = \int L(X)\,\mathrm{d}X \tag{2}$$

$$\mathrm{d}X = \pi(\theta)\mathrm{d}\theta$$

The prior mass can be accumulated from its elements ($\mathrm{d}X$) in any order. Briefly, the cumulant mass of likelihood $> \lambda$ can be defined (3), and this allows the evidence to be written as a one-dimensional integral of the (inverse) likelihood $L(X)$ over the unit range (taking the enclosed prior mass X to be the primary variable) (4) (Skilling 2006).

$$X(\lambda) = \int_{L(\theta)>\lambda} \pi(\theta)\,\mathrm{d}\theta \tag{3}$$

$$Z = \int_0^1 L(X)\,\mathrm{d}X \tag{4}$$

$$L(X(\lambda)) \equiv \lambda$$

Given a sequence of decreasing values $0 < X_m < \ldots X_2 < X_1 < 1$ where the likelihood $L_i = L(X_i)$ can be evaluated, the evidence can be approximated numerically as a weighted sum (5).

$$Z = \sum_{i=1}^{m} L_i w_i \tag{5}$$

$$w_i = \Delta X_i$$

Inferences about the posterior can be obtained from the sequence of m discarded points generated by sampling, P. Each point is assigned the weight $p_i = L(\theta_i)w_i/Z$, and the first and second moments of the jth parameter in the vector θ can be estimated by (6) and (7) respectively. Nested sampling was run with 50 active samples in the analysis reported here. The algorithm is scalable to at least 30 dimensions and has only a single configuration parameter (the number of active samples). Further details and R code can be found in (Aitken and Akman 2013).

$$\langle \theta_j \rangle = \sum_{i=1}^{m} p_i \theta_{ij} \tag{6}$$

$$\mathrm{var}\ \theta_j = \left(\sum_{i=1}^{m} p_i (\theta_{ij})^2 \right) - \langle \theta_j \rangle^2 \tag{7}$$

References

Aitken, S. and Akman, O. (2013). Nested sampling for parameter inference in systems biology: application to an exemplar circadian model. *BMC Systems Biology*, **7**(1), 72.

Arner, E., Daub, C. O., Vitting-Seerup, K., Andersson, R., Lilje, B., Drablos, F., Lennartsson, A., Rönnerblad, M., Hrydziuszsko, O., Vitezic, M., FANTOM Consortium, and the Riken Omics Science Center (2015). Enhancers lead waves of coordinated transcription in transitioning mammalian cells. *Science*, **-**(-), –.

Eden, E., Navon, R., Steinfeld, I., Lipson, D., and Yakhini, Z. (2009). GOrilla: a tool for discovery and visualization of enriched GO terms in ranked gene lists. *BMC Bioinformatics*, **10**(1), 48.

Skilling, J. (2006). Nested sampling for general Bayesian computations. *Bayesian Analysis*, **1**(4), 833–860.

Supek, F., Bosnjak, M., Skunca, N., and Smuc, T. (2011). REVIGO summarizes and visualizes long lists of gene ontology terms. *PLoS ONE*, **6**(7), e21800.
