# Supplementary figures and images for: Transcriptional Dynamics Reveal Critical Roles for Non-coding RNAs in the Immediate-Early Response
Source: PLoS Comput Biol. 2015 Apr 17;11(4):e1004217. doi: 10.1371/journal.pcbi.1004217 (PMC4401570; doi:10.1371/journal.pcbi.1004217)

**AoSMC-FGF2 (23% assigned)**

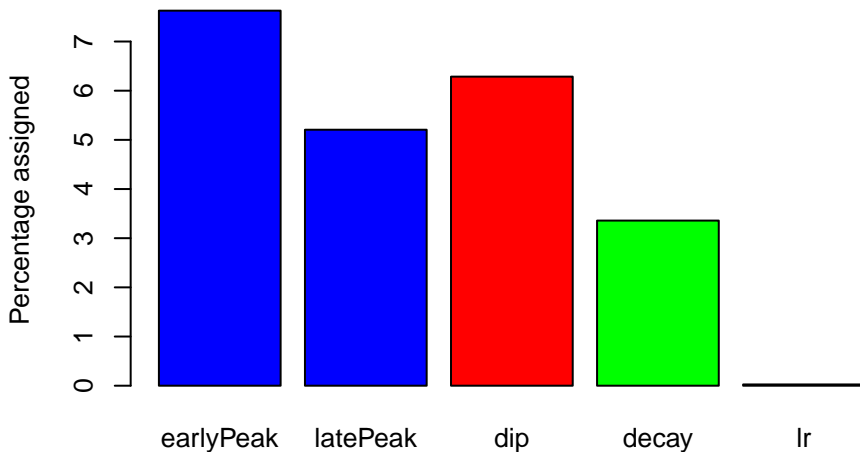

**AoSMC-IL1b (21% assigned)**

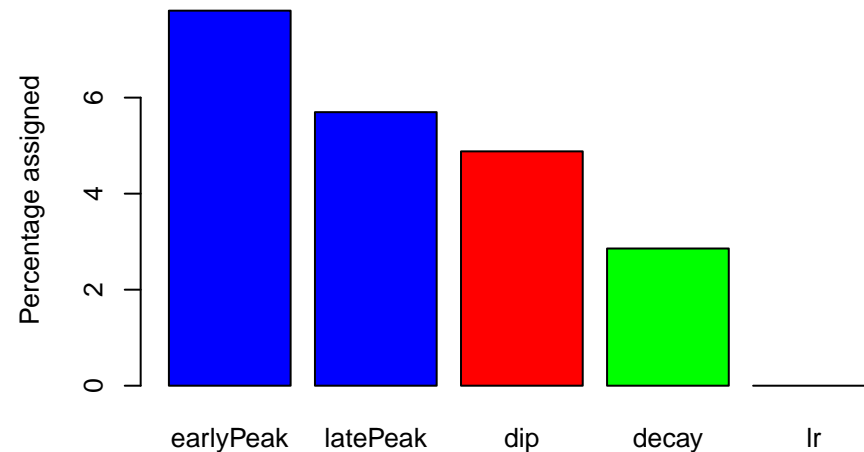

**MCF7-EGF (33% assigned)**

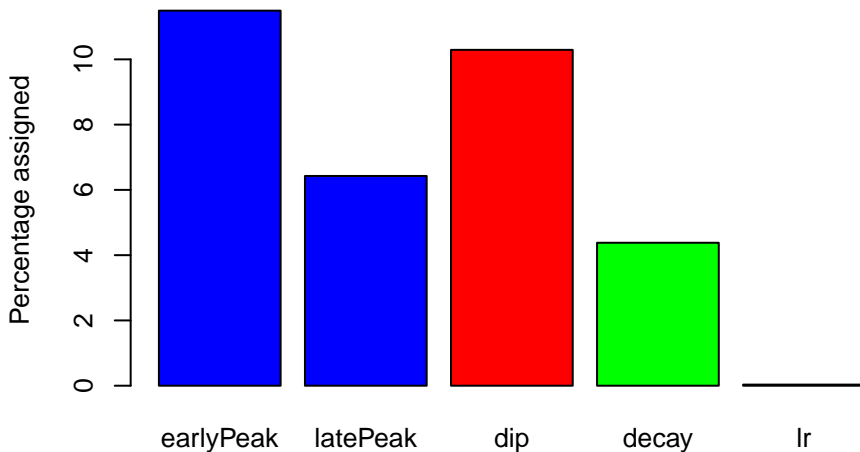

**MCF7-HRG (33% assigned)**

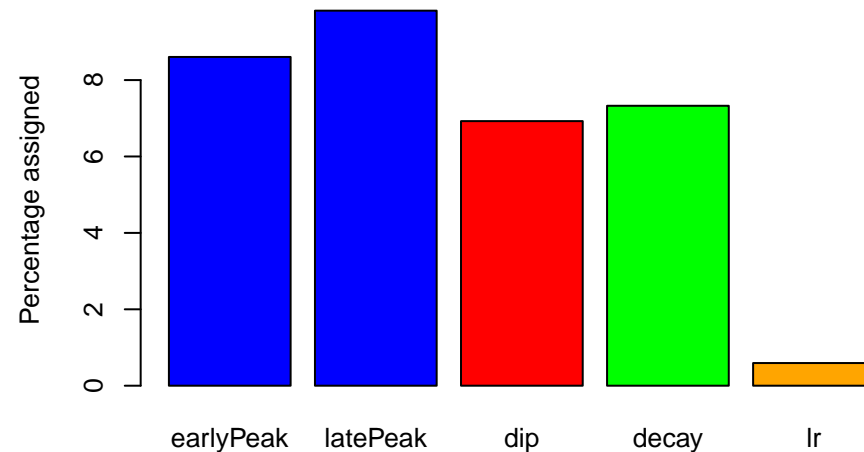

Supplement: S1 Fig — (PDF) [file pcbi.1004217.s002.pdf]

**AoSMC-FGF2 (30% assigned)**

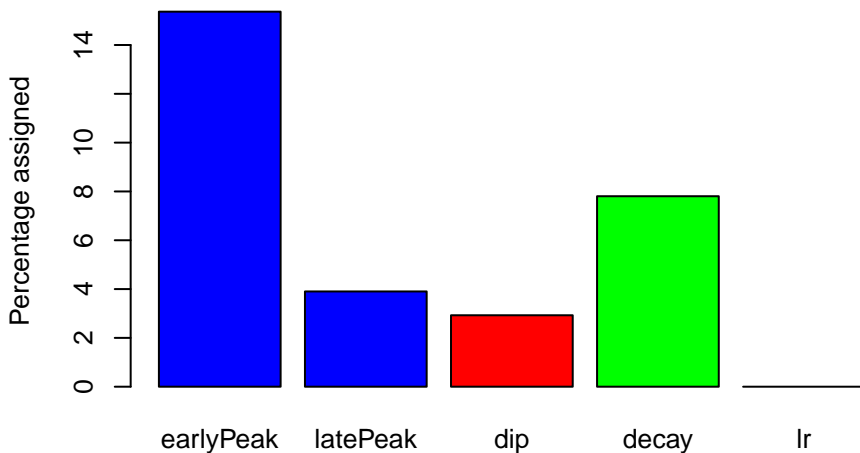

**AoSMC-IL1b (31% assigned)**

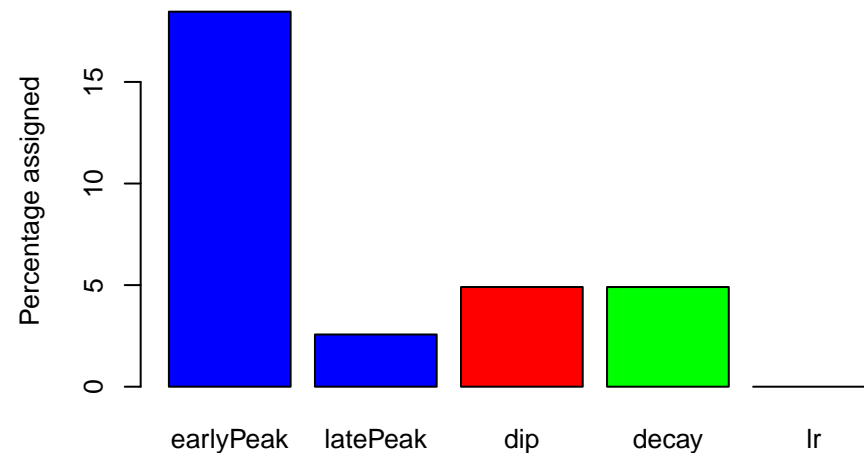

**MCF7-EGF (36% assigned)**

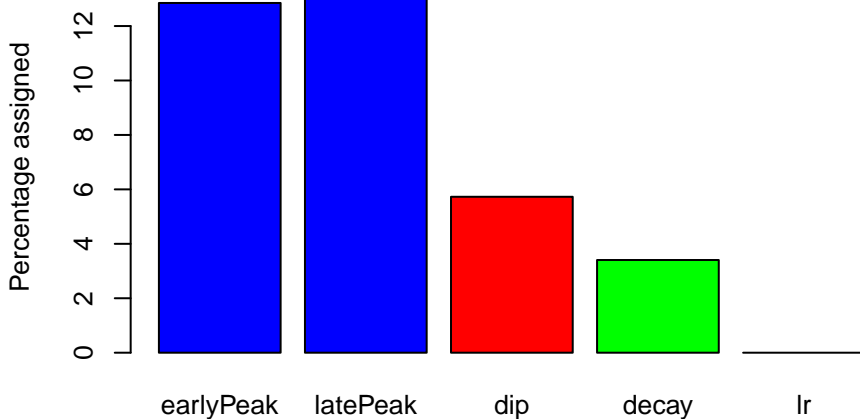

**MCF7-HRG (39% assigned)**

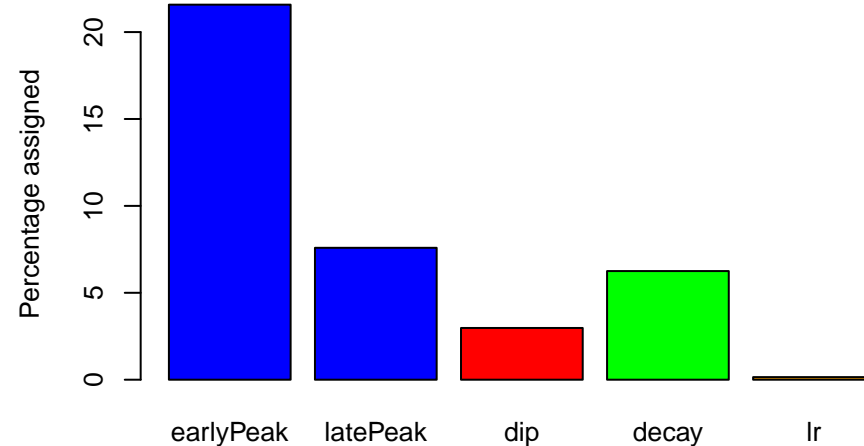

Supplement: S2 Fig — (PDF) [file pcbi.1004217.s003.pdf]

**LZIC MCF7-HRG [NODECISION]**

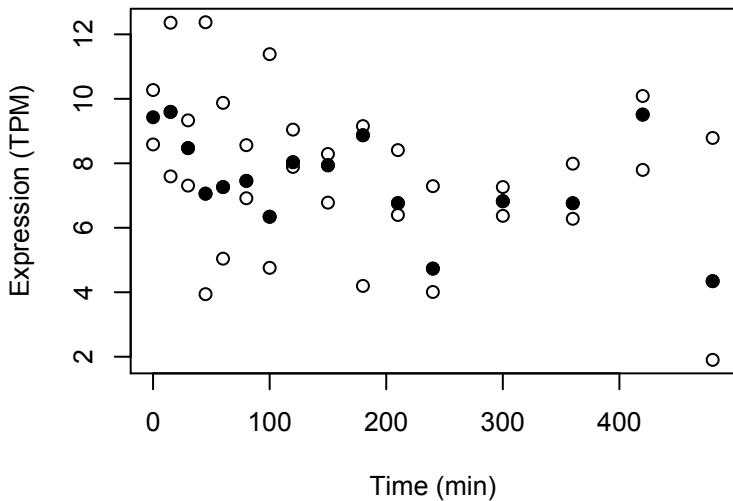

**ATP1B1 MCF7-HRG [latePeak]**

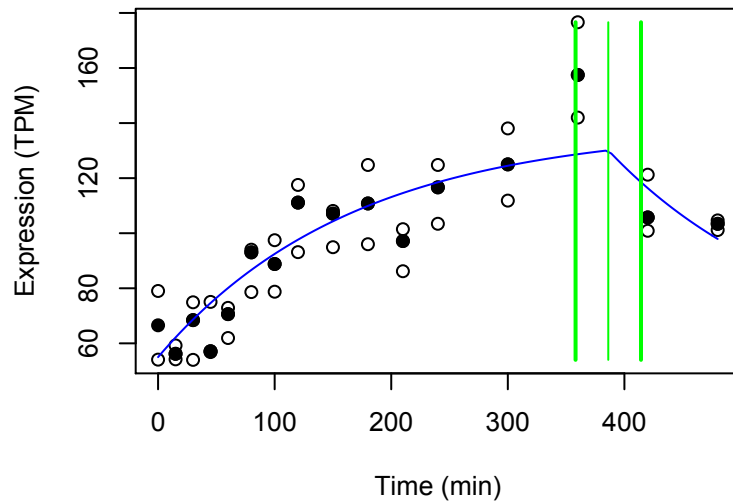

**CEPT1 MCF7-HRG [dip]**

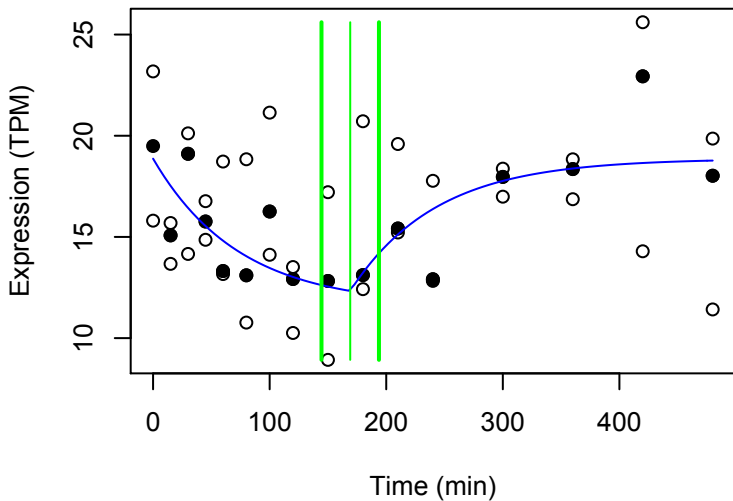

**EXOSC10 MCF7-HRG [decay]**

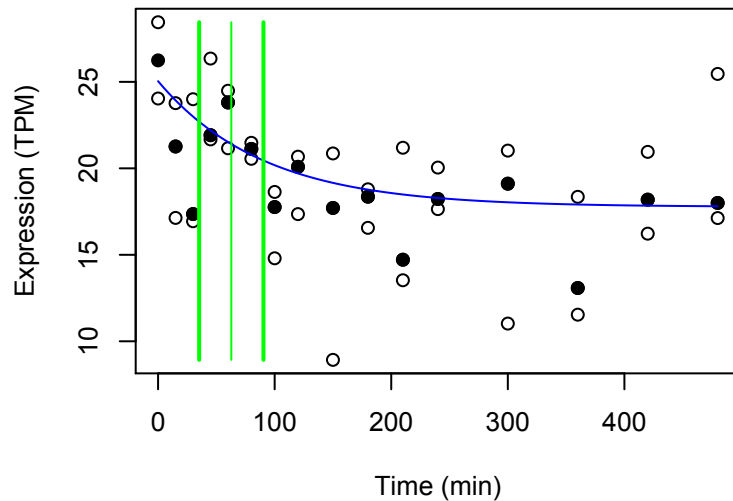

Supplement: S3 Fig — CAGE TPM values are plotted as circles (median value is filled), predictions of the kinetic signature models using parameter means are shown in blue and the vertical green lines indicate the mean t S (or t h in the case of the decay signature) and one standard deviation above and below. (PDF) [file pcbi.1004217.s004.pdf]

REVIGO Gene Ontology treemap

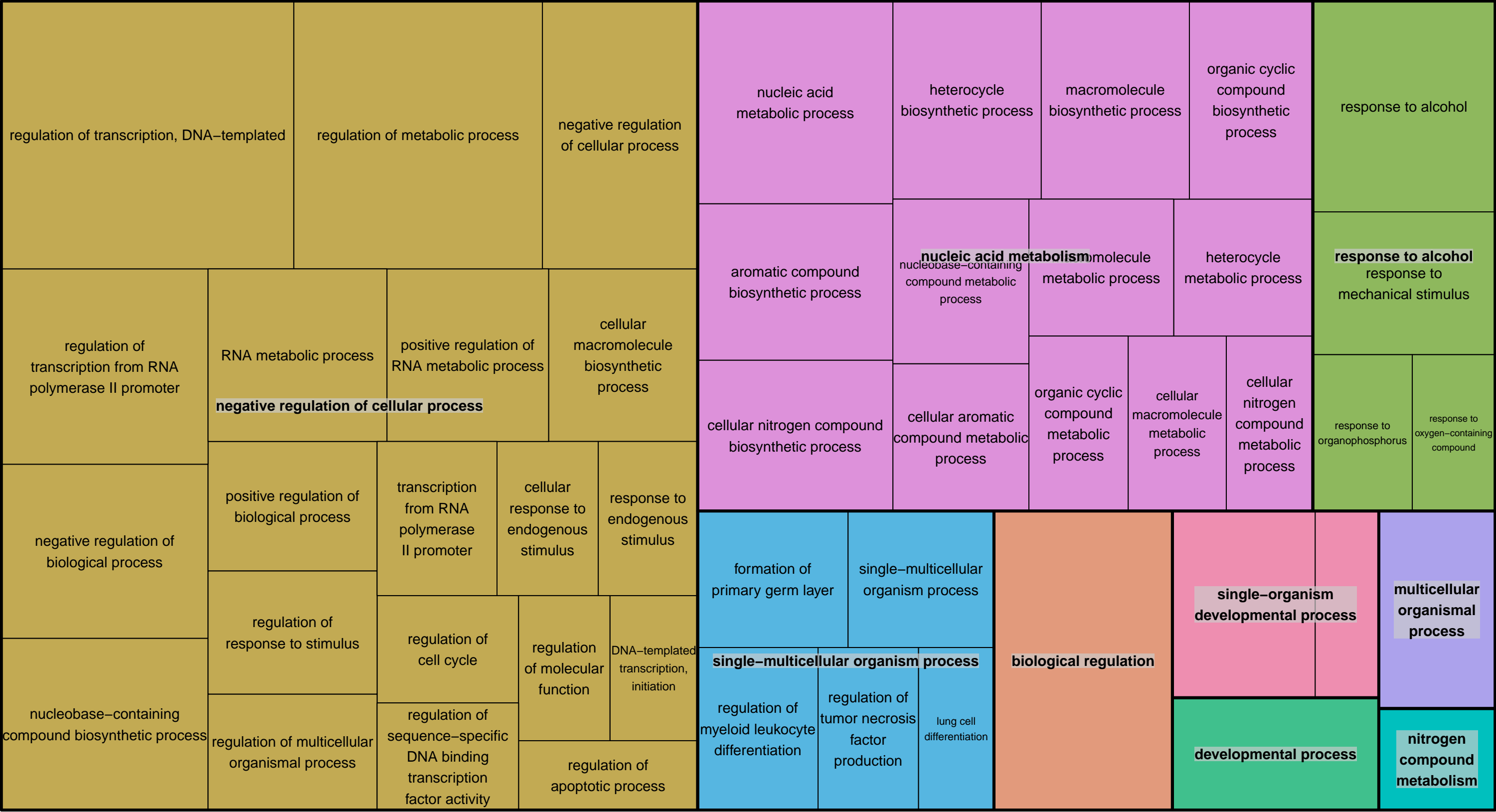

Supplement: S6 Fig — This analysis was performed with GOrilla [22] and REVIGO [23]. (PDF) [file pcbi.1004217.s007.pdf]

**Early peak**

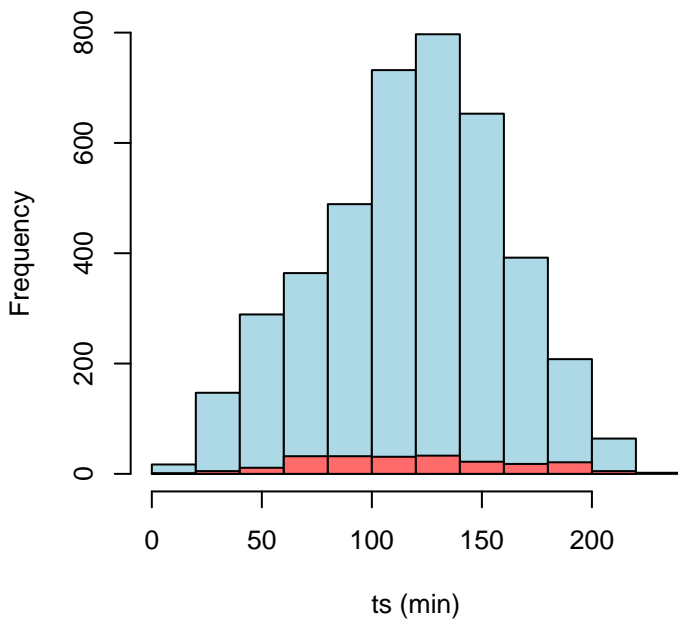

**Late peak**

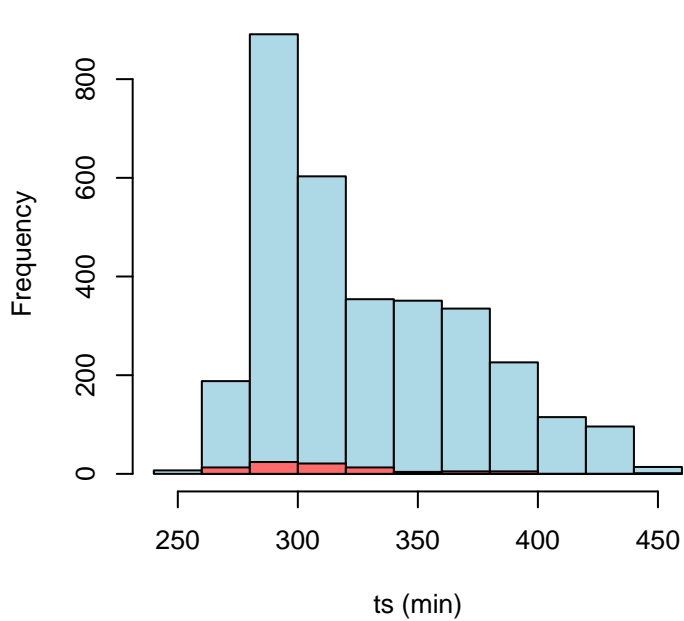

**Dip**

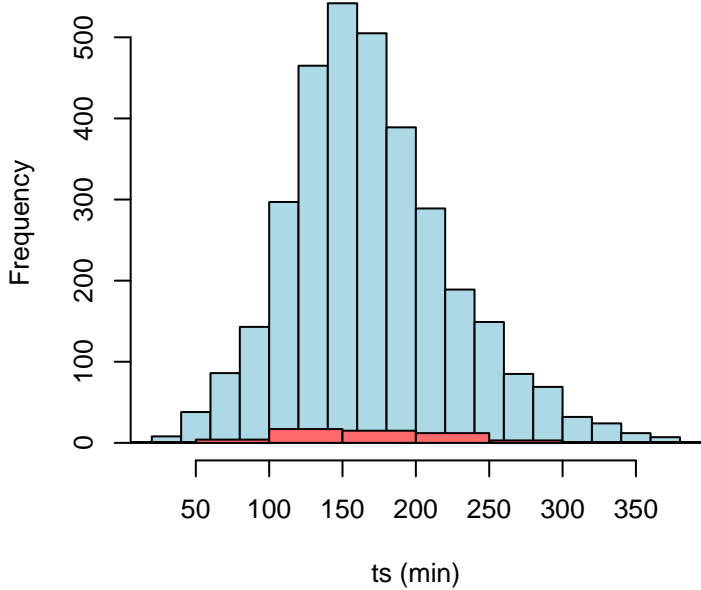

**Decay**

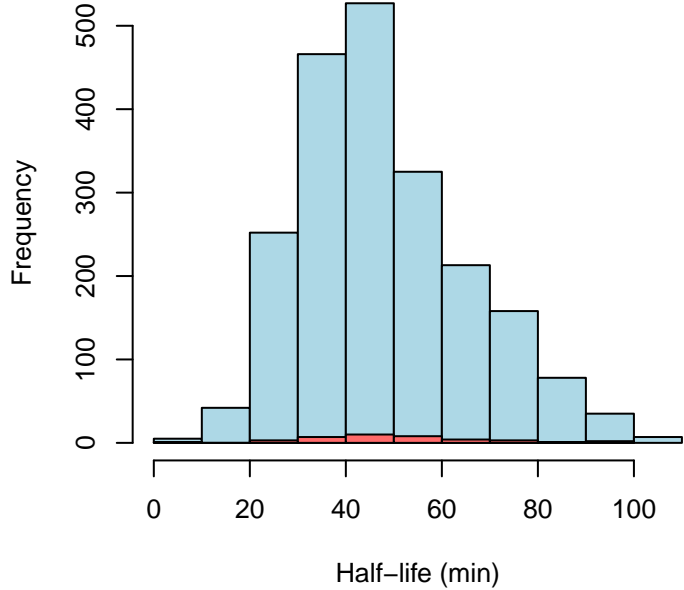

Supplement: S7 Fig — The distributions of t s for early peak, late peak, dip signatures and the half-life of the decay signature are shown in blue, the distributions of the subsets of known IEGs in each category are shown in red. The apparent bimodal distibution of t s for peak models is an artefact of the different choices of prior range. When a peak model is run without the early or late restriction the distribution of switch times is not bimodal, however, fewer time courses are assigned to the peak category. (PDF) [file pcbi.1004217.s008.pdf]

B

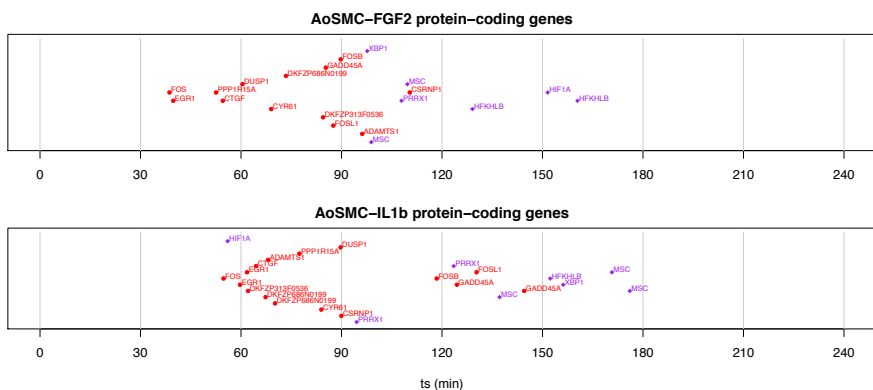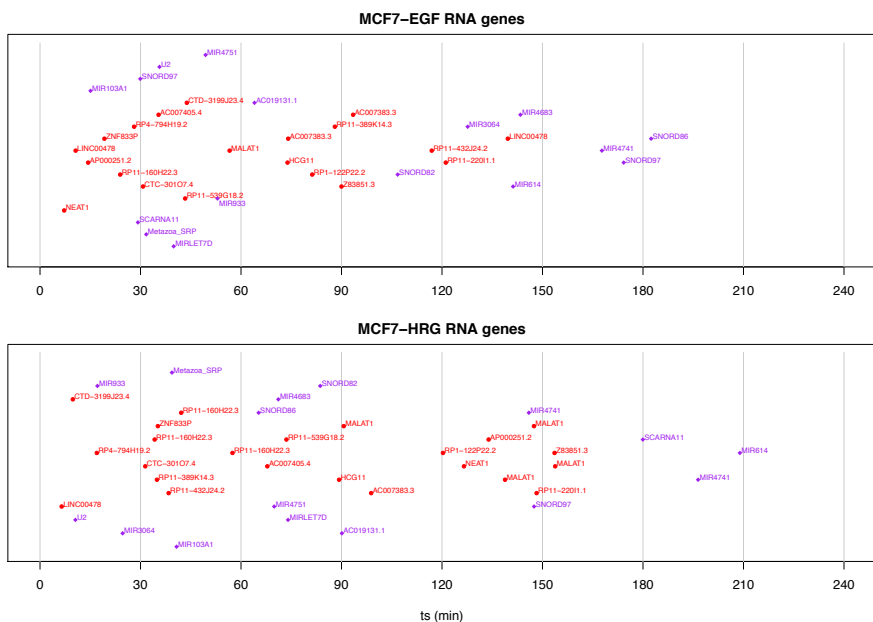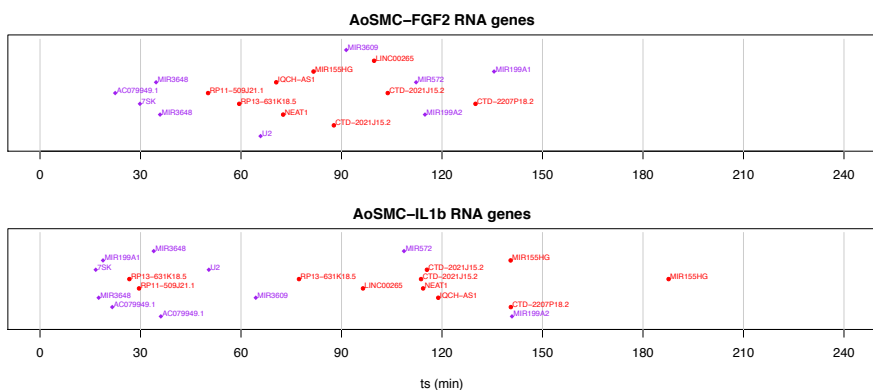

Supplement: S8 Fig — (A) The timing of known IEGs and transcription factors is shown for IEGs (red) and TFs (blue) assigned to the early peak signature in each AoSMC data set. (B) The timing of non-coding genes assigned to the early peak category is shown for lncRNA (red) and all other ncRNA (blue) in MCF7 data and (C) in AoSMC data. Symbols indicate the t s (x axis) and are labelled with the gene name associated with the CAGE cluster. (PDF) [file pcbi.1004217.s009.pdf]

A

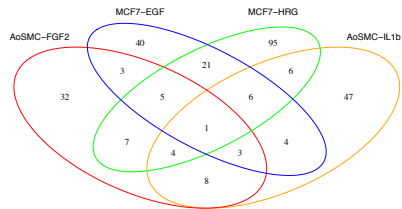

B

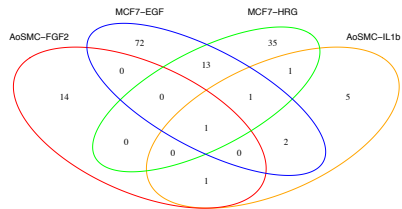

C

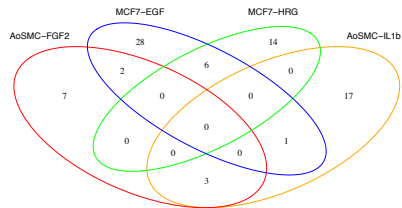

D

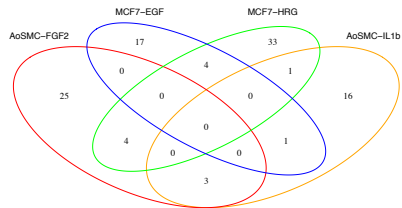

Supplement: S9 Fig — (A) early peak; (B) late peak; (C) dip; (D) decay. (PDF) [file pcbi.1004217.s010.pdf]

Mature hsa-mir-10b [decay]

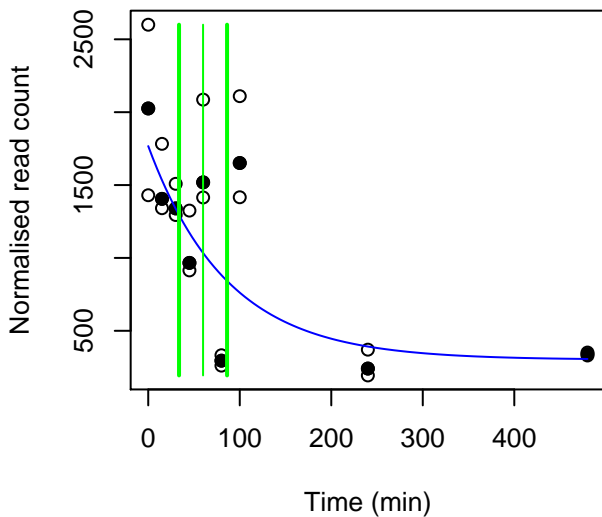

Mature hsa-mir-146a [decay]

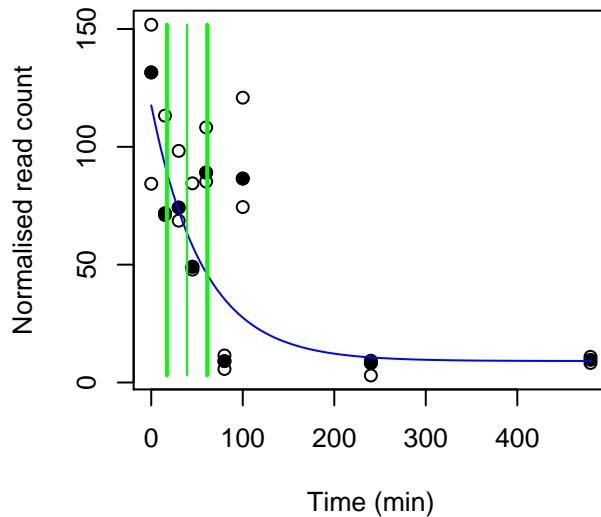

Mature hsa-mir-522 [dip]

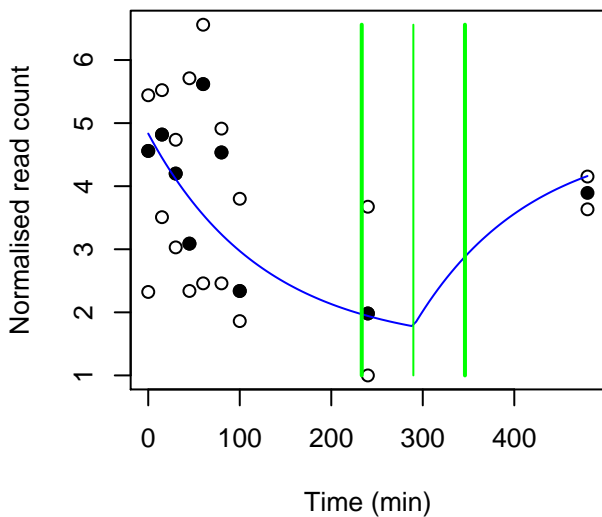

Mature hsa-mir-937 [dip]

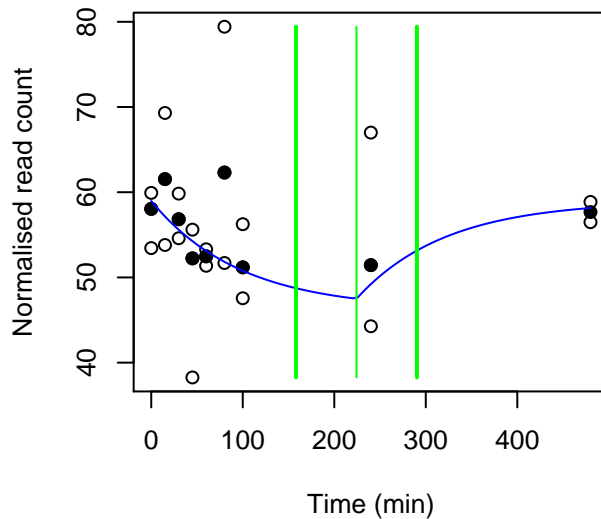

Supplement: S13 Fig — The data and kinetic signatures are presented for two mature miRNA assigned to the decay signature and for two miRNA assigned to the dip signature. Expression values are plotted as circles (median value is filled), predictions of the model using parameter means are shown in blue and the vertical green lines indicate the mean t h (or t S) and one standard deviation above and below. (PDF) [file pcbi.1004217.s014.pdf]
