# Supplementary material for: Transcriptional Dynamics Reveal Critical Roles for Non-coding RNAs in the Immediate-Early Response
Source: PLoS Comput Biol. 2015 Apr 17;11(4):e1004217. doi: 10.1371/journal.pcbi.1004217 (PMC4401570; doi:10.1371/journal.pcbi.1004217)
Supplement: S4 Fig — (A) early peak; (B) late peak; (C) dip; (D) decay. (PDF) [file pcbi.1004217.s005.pdf]

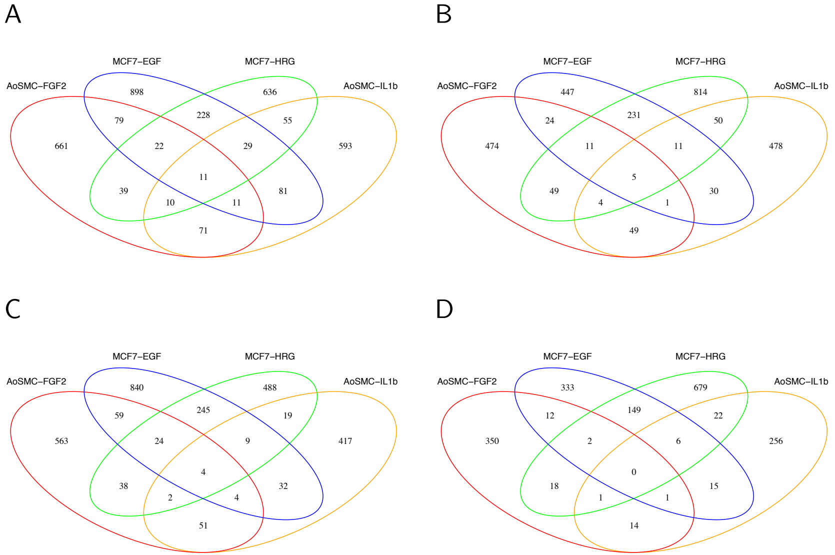
A
MCF7−EGF
MCF7−HRG
AoSMC−FGF2
AoSMC−IL1b
898
636
79
228
55
661
22
29
593
11
39
81
10
11
71
B
MCF7−EGF
MCF7−HRG
AoSMC−FGF2
AoSMC−IL1b
447
814
24
231
50
474
11
11
478
5
49
30
4
1
49
C
MCF7−EGF
MCF7−HRG
AoSMC−FGF2
AoSMC−IL1b
840
488
59
245
19
563
24
9
417
4
38
32
2
4
51
D
MCF7−EGF
MCF7−HRG
AoSMC−FGF2
AoSMC−IL1b
333
679
12
149
22
350
2
6
256
0
18
15
1
1
14
