# Supplementary material for: Transcriptional Dynamics Reveal Critical Roles for Non-coding RNAs in the Immediate-Early Response
Source: PLoS Comput Biol. 2015 Apr 17;11(4):e1004217. doi: 10.1371/journal.pcbi.1004217 (PMC4401570; doi:10.1371/journal.pcbi.1004217)
Supplement: S5 Fig — CAGE TPM values are plotted as circles (median value is filled), predictions of the kinetic signature models using parameter means are shown in blue and the vertical green lines indicate the mean t S and one standard deviation above and below. (PDF) [file pcbi.1004217.s006.pdf]

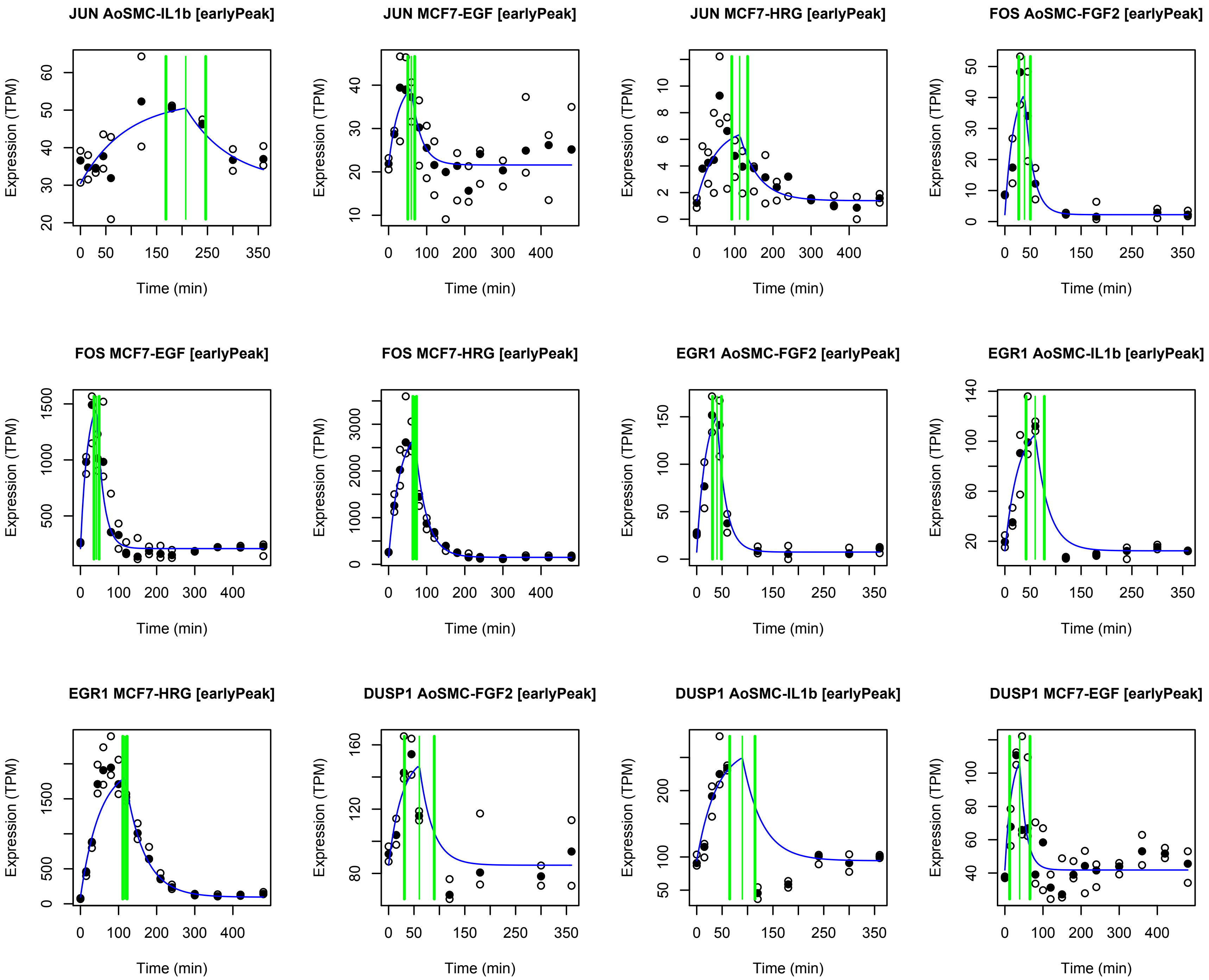
JUN AoSMC-IL1b [earlyPeak]
JUN MCF7-EGF [earlyPeak]
JUN MCF7-HRG [earlyPeak]
FOS AoSMC-FGF2 [earlyPeak]
FOS MCF7-EGF [earlyPeak]
FOS MCF7-HRG [earlyPeak]
EGR1 AoSMC-FGF2 [earlyPeak]
EGR1 AoSMC-IL1b [earlyPeak]
EGR1 MCF7-HRG [earlyPeak]
DUSP1 AoSMC-FGF2 [earlyPeak]
DUSP1 AoSMC-IL1b [earlyPeak]
DUSP1 MCF7-EGF [earlyPeak]
Expression (TPM)
Time (min)
