# Supplementary material for: Transcriptional Dynamics Reveal Critical Roles for Non-coding RNAs in the Immediate-Early Response
Source: PLoS Comput Biol. 2015 Apr 17;11(4):e1004217. doi: 10.1371/journal.pcbi.1004217 (PMC4401570; doi:10.1371/journal.pcbi.1004217)
Supplement: S10 Fig — CAGE TPM values are plotted as circles (median value is filled), predictions of the kinetic signature models using parameter means are shown in blue and the vertical green lines indicate the mean t S and one standard deviation above and below. (PDF) [file pcbi.1004217.s011.pdf]

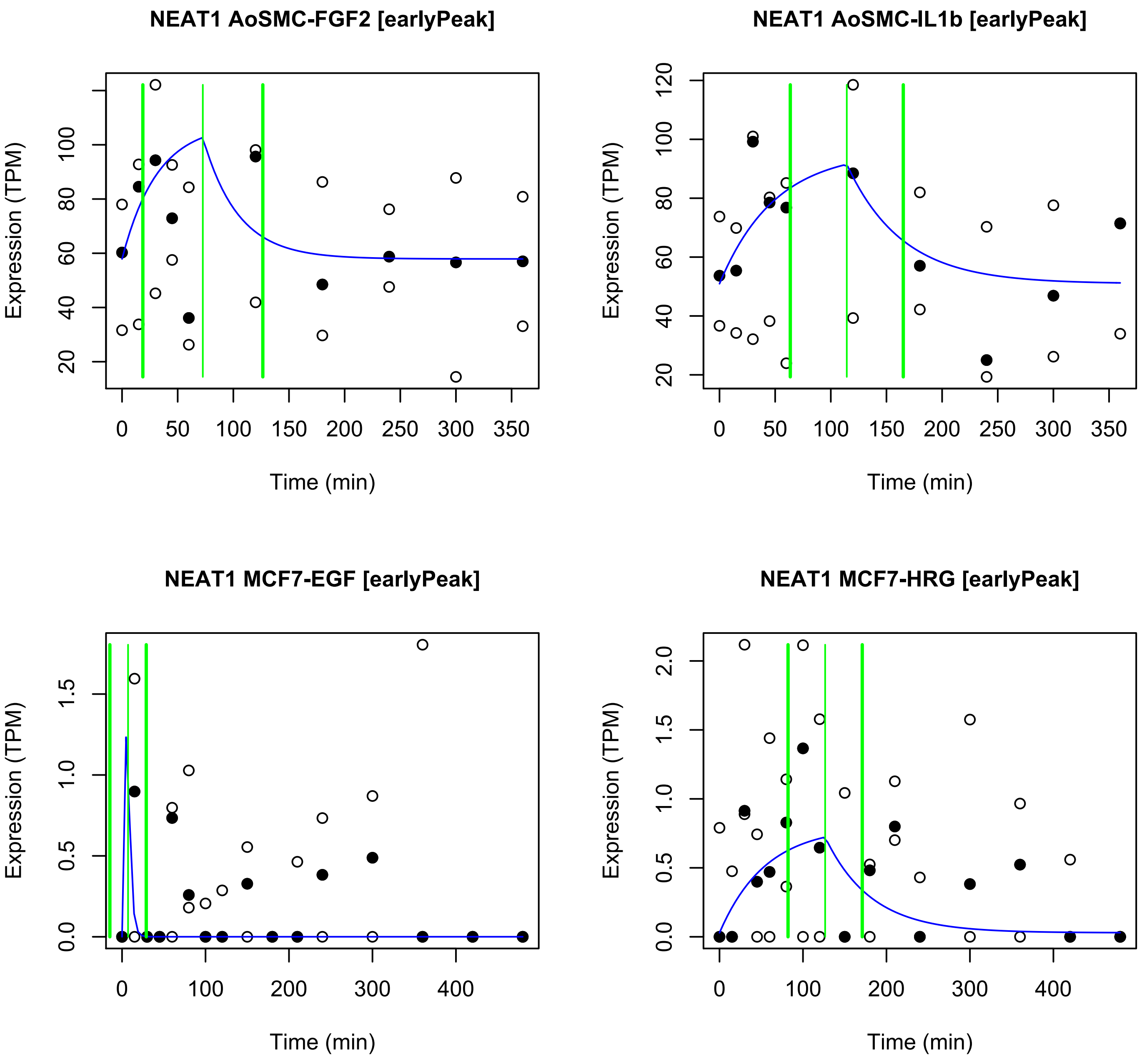
NEAT1 AoSMC-FGF2 [earlyPeak]
NEAT1 AoSMC-IL1b [earlyPeak]
NEAT1 MCF7-EGF [earlyPeak]
NEAT1 MCF7-HRG [earlyPeak]
Expression (TPM)
Time (min)
20
40
60
80
100
120
0
50
100
150
200
250
300
350
0.0
0.5
1.0
1.5
2.0
400
