# Supplementary material for: Transcriptional Dynamics Reveal Critical Roles for Non-coding RNAs in the Immediate-Early Response
Source: PLoS Comput Biol. 2015 Apr 17;11(4):e1004217. doi: 10.1371/journal.pcbi.1004217 (PMC4401570; doi:10.1371/journal.pcbi.1004217)
Supplement: S11 Fig — (Top) Distributions of early peak DNaseI counts (blue) and non-early peak counts (black) for protein-coding CAGE clusters, and QQ plot. Early peak clusters have significantly higher counts. (Middle) Distributions of early peak DNaseI counts (blue) and non-early peak counts (black) for non-coding CAGE clusters, and QQ plot. There is no significant difference between the distributions. (Bottom) Distributions DNaseI counts for early peak lncRNA (blue) and all other counts (black) for non-coding CAGE clusters, and QQ plot. Early peak lncRNA clusters have significantly higher counts. (PDF) [file pcbi.1004217.s012.pdf]

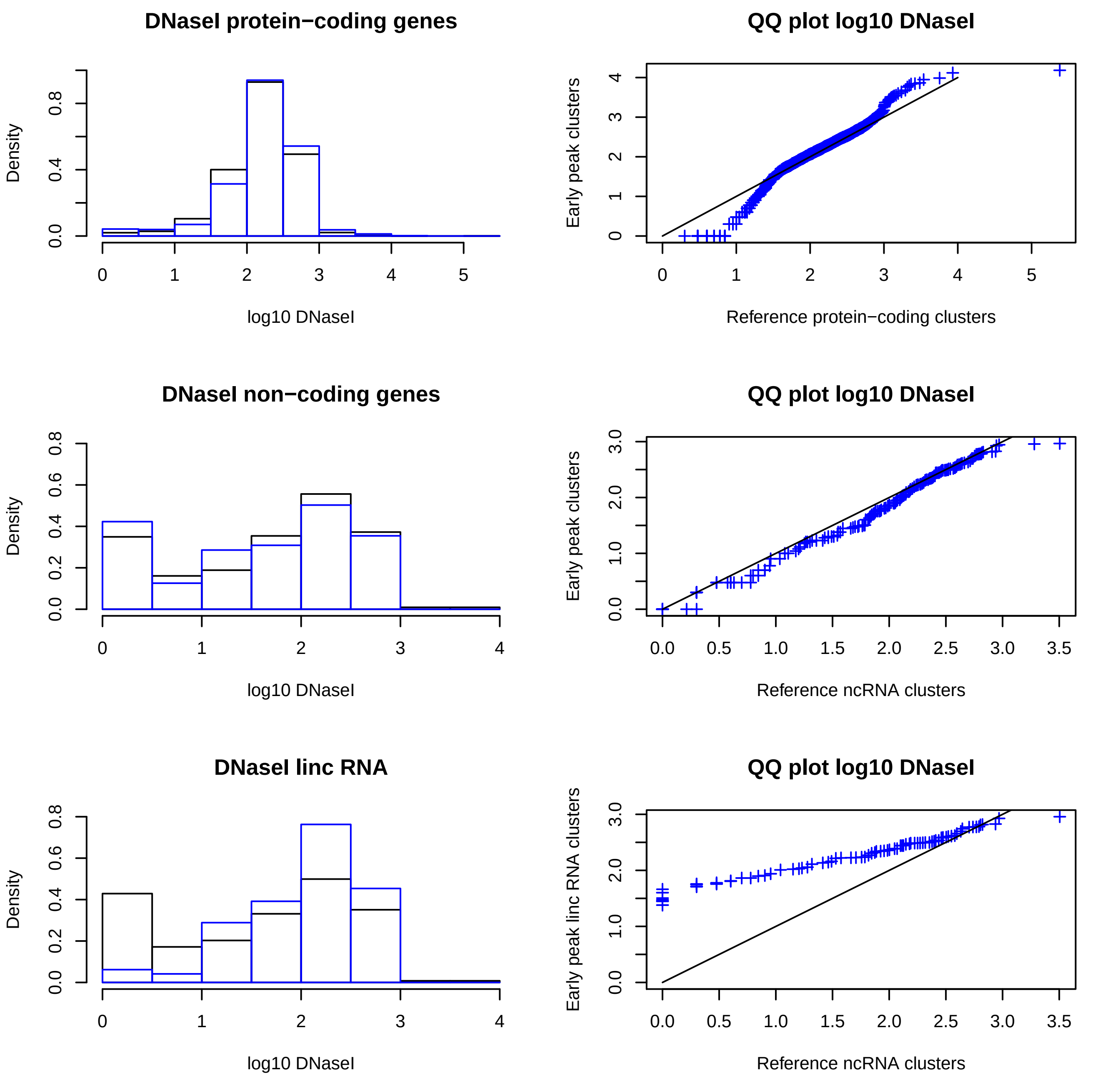

DNaseI protein−coding genes
Density
0.0
0.4
0.8
0
1
2
3
4
5
log10 DNaseI
QQ plot log10 DNaseI
Early peak clusters
0
1
2
3
4
0
1
2
3
4
5
Reference protein−coding clusters
DNaseI non−coding genes
Density
0.0
0.2
0.4
0.6
0.8
0
1
2
3
4
log10 DNaseI
QQ plot log10 DNaseI
Early peak clusters
0.0
1.0
2.0
3.0
0.0
0.5
1.0
1.5
2.0
2.5
3.0
3.5
Reference ncRNA clusters
DNaseI linc RNA
Density
0.0
0.2
0.4
0.6
0.8
0
1
2
3
4
log10 DNaseI
QQ plot log10 DNaseI
Early peak linc RNA clusters
0.0
1.0
2.0
3.0
0.0
0.5
1.0
1.5
2.0
2.5
3.0
3.5
Reference ncRNA clusters
