# Supplementary material for: Transcriptional Dynamics Reveal Critical Roles for Non-coding RNAs in the Immediate-Early Response
Source: PLoS Comput Biol. 2015 Apr 17;11(4):e1004217. doi: 10.1371/journal.pcbi.1004217 (PMC4401570; doi:10.1371/journal.pcbi.1004217)
Supplement: S12 Fig — Data is presented for the host lncRNA of hsa-mir-155 (MIR155HG ENSG00000234883) in AoSMC-FGF2, AoSMC-Il1b and MCF7-HRG data sets (data for MCF7-EGF does not pass quality controls). CAGE TPM values are plotted as circles (median value is filled), predictions of the kinetic signature models using parameter means are shown in blue and the vertical green lines indicate the mean t S and one standard deviation above and below. (PDF) [file pcbi.1004217.s013.pdf]

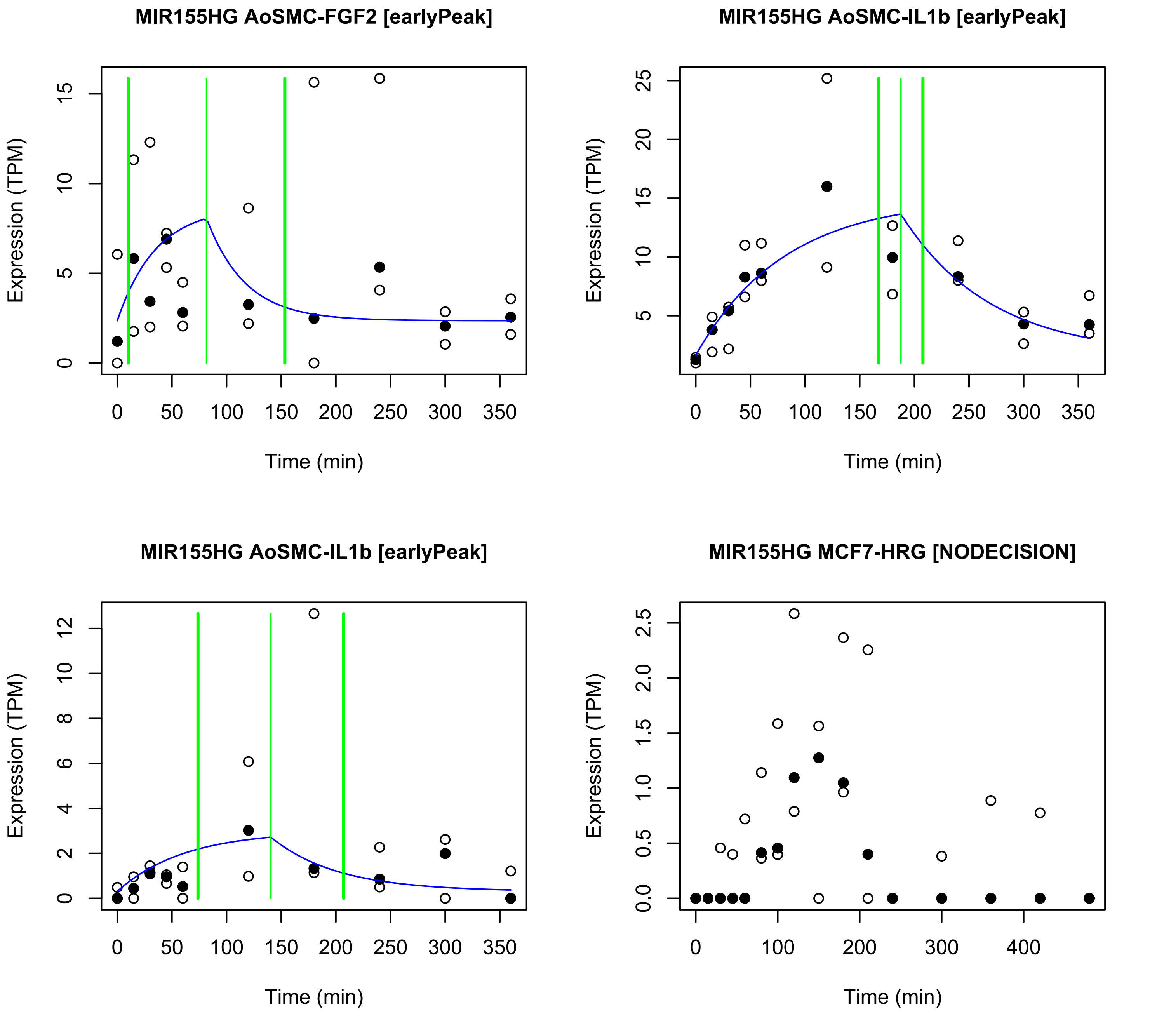
MIR155HG AoSMC-FGF2 [earlyPeak]
MIR155HG AoSMC-IL1b [earlyPeak]
MIR155HG AoSMC-IL1b [earlyPeak]
MIR155HG MCF7-HRG [NODECISION]
Expression (TPM)
Time (min)
