# Supplementary material for: Transcriptional Dynamics Reveal Critical Roles for Non-coding RNAs in the Immediate-Early Response
Source: PLoS Comput Biol. 2015 Apr 17;11(4):e1004217. doi: 10.1371/journal.pcbi.1004217 (PMC4401570; doi:10.1371/journal.pcbi.1004217)
Supplement: S14 Fig — Eleven ID-miRs were present in the small RNA sequencing data with expression above the minimum threshold. Expression values are plotted as circles (median value is filled), and the dashed purple lines indicate the best-fitting kinetic signature. None of the ID-miR assignments passed the standard statistical criteria, hence these assignments are not significant (NS) and plotted for information only. It is apparent that hsa-mir-155 increases linearly, and that hsa-mir-191 peaks early in the time course. A number of ID-miRs show the expected immediate downregulation including hsa-mir-212 and hsa-mir-320a. (PDF) [file pcbi.1004217.s015.pdf]

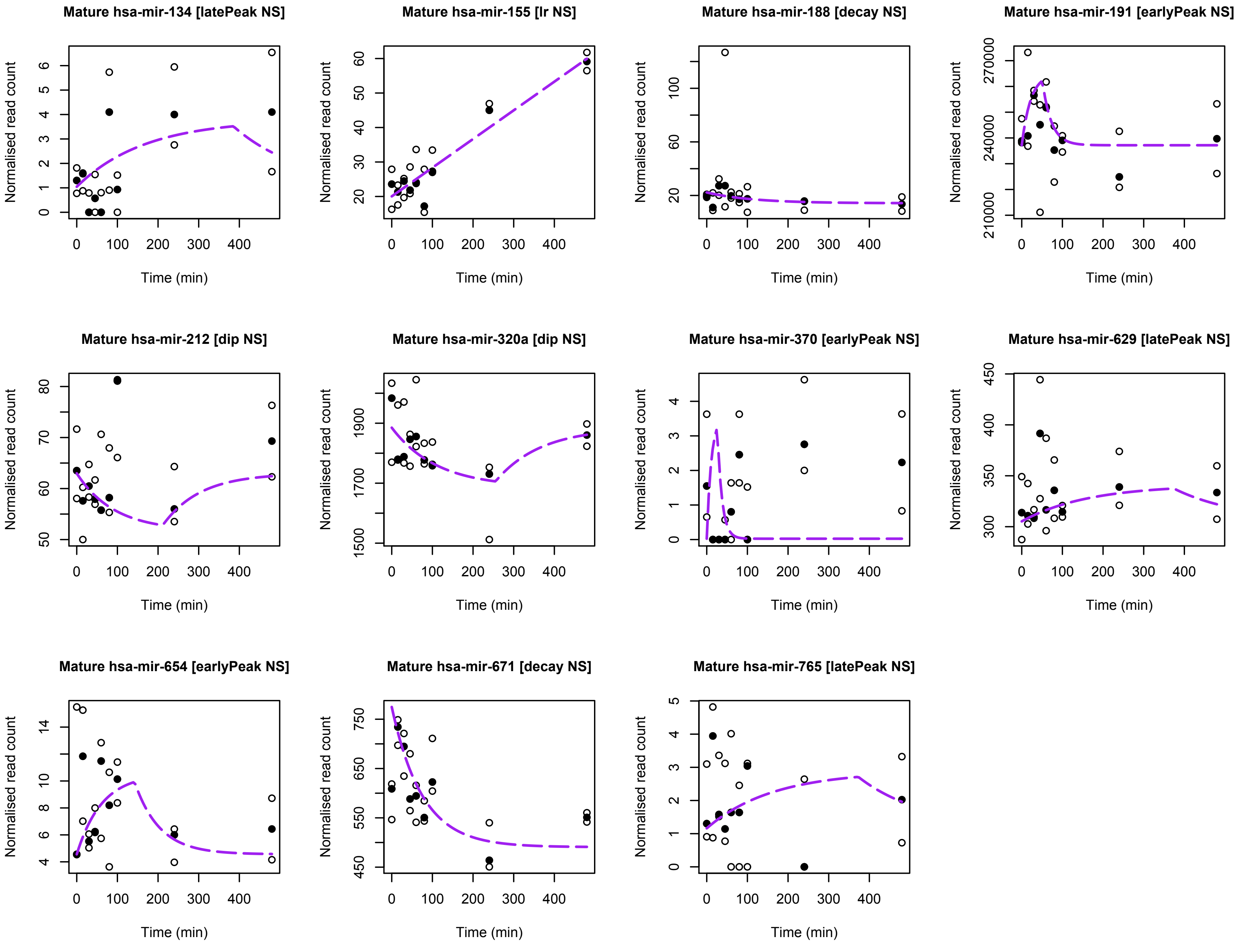
Mature hsa-mir-134 [latePeak NS]
Mature hsa-mir-155 [lr NS]
Mature hsa-mir-188 [decay NS]
Mature hsa-mir-191 [earlyPeak NS]
Mature hsa-mir-212 [dip NS]
Mature hsa-mir-320a [dip NS]
Mature hsa-mir-370 [earlyPeak NS]
Mature hsa-mir-629 [latePeak NS]
Mature hsa-mir-654 [earlyPeak NS]
Mature hsa-mir-671 [decay NS]
Mature hsa-mir-765 [latePeak NS]
Normalised read count
Time (min)
0 100 200 300 400
0 1 2 3 4 5 6
20 30 40 50 60
20 60 100
210000 240000 270000
50 60 70 80
1500 1700 1900
0 1 2 3 4
300 350 400 450
4 6 8 10 14
450 550 650 750
0 1 2 3 4 5
