# Supplementary material for: Transcriptional Dynamics Reveal Critical Roles for Non-coding RNAs in the Immediate-Early Response
Source: PLoS Comput Biol. 2015 Apr 17;11(4):e1004217. doi: 10.1371/journal.pcbi.1004217 (PMC4401570; doi:10.1371/journal.pcbi.1004217)
Supplement: S15 Fig — Median CAGE expression (black circles) of precursor miRNA and median mature miRNA expression (red triangles). Lines are are a spline fitted to the data. (PDF) [file pcbi.1004217.s016.pdf]

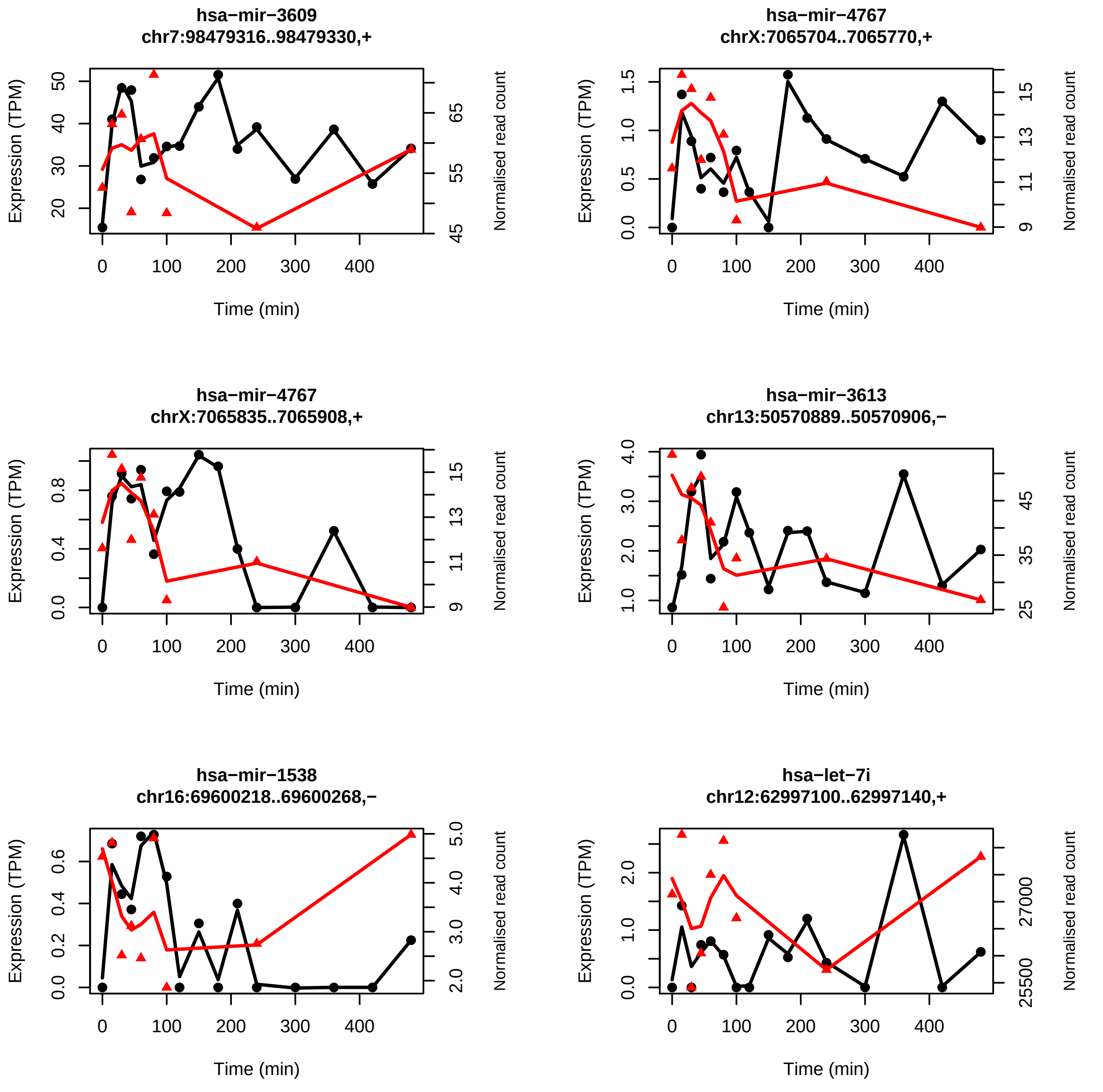

hsa−mir−3609
chr7:98479316..98479330,+
hsa−mir−4767
chrX:7065704..7065770,+
hsa−mir−4767
chrX:7065835..7065908,+
hsa−mir−3613
chr13:50570889..50570906,−
hsa−mir−1538
chr16:69600218..69600268,−
hsa−let−7i
chr12:62997100..62997140,+
Expression (TPM)
Normalised read count
Time (min)

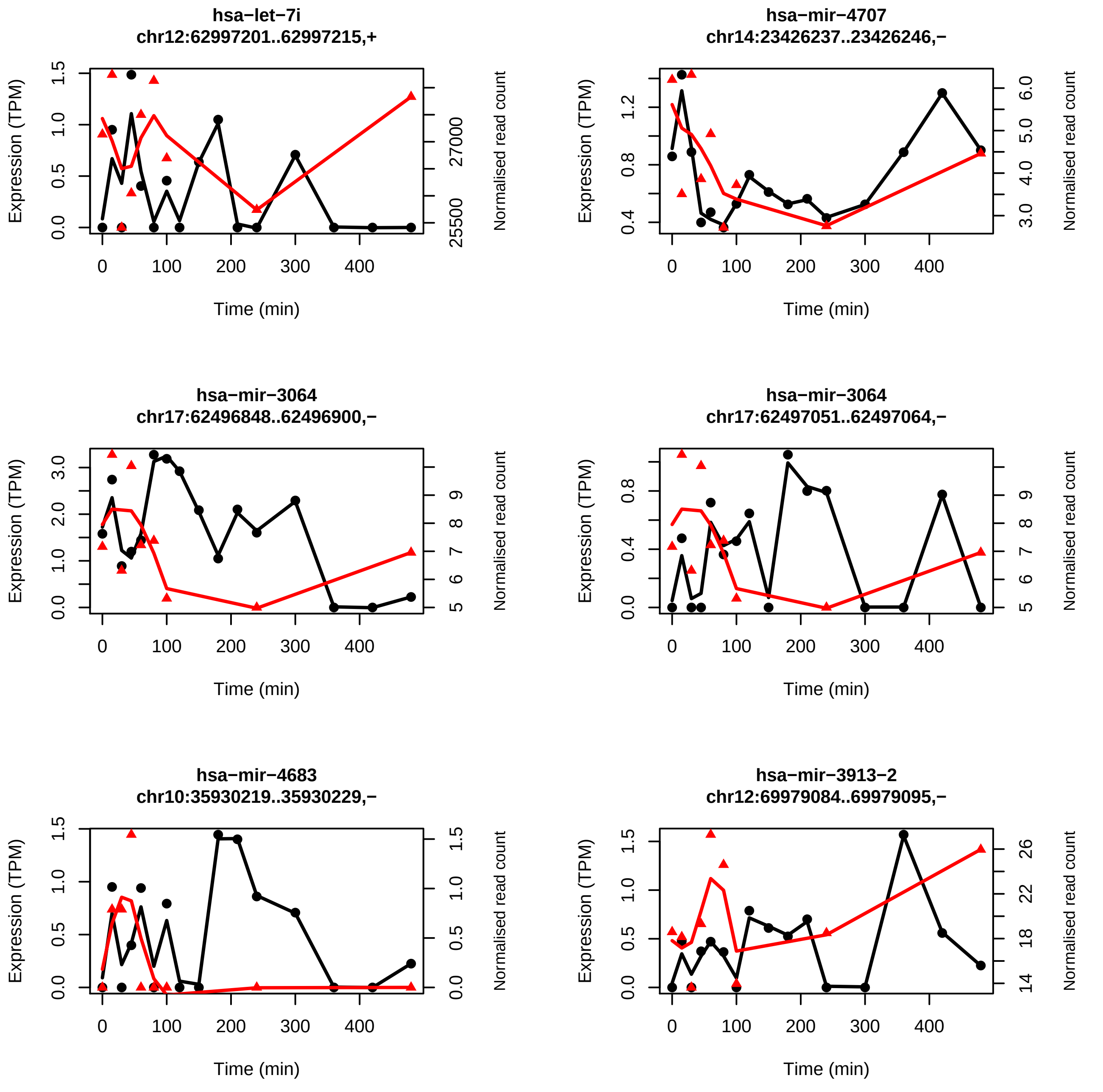
hsa−let−7i
chr12:62997201..62997215,+
Expression (TPM)
Normalised read count
Time (min)
0.0 0.5 1.0 1.5
25500 27000
0 100 200 300 400
hsa−mir−4707
chr14:23426237..23426246,−
Expression (TPM)
Normalised read count
Time (min)
0.4 0.8 1.2
3.0 4.0 5.0 6.0
0 100 200 300 400
hsa−mir−3064
chr17:62496848..62496900,−
Expression (TPM)
Normalised read count
Time (min)
0.0 1.0 2.0 3.0
5 6 7 8 9
0 100 200 300 400
hsa−mir−3064
chr17:62497051..62497064,−
Expression (TPM)
Normalised read count
Time (min)
0.0 0.4 0.8
5 6 7 8 9
0 100 200 300 400
hsa−mir−4683
chr10:35930219..35930229,−
Expression (TPM)
Normalised read count
Time (min)
0.0 0.5 1.0 1.5
0.0 0.5 1.0 1.5
0 100 200 300 400
hsa−mir−3913−2
chr12:69979084..69979095,−
Expression (TPM)
Normalised read count
Time (min)
0.0 0.5 1.0 1.5
14 18 22 26
0 100 200 300 400

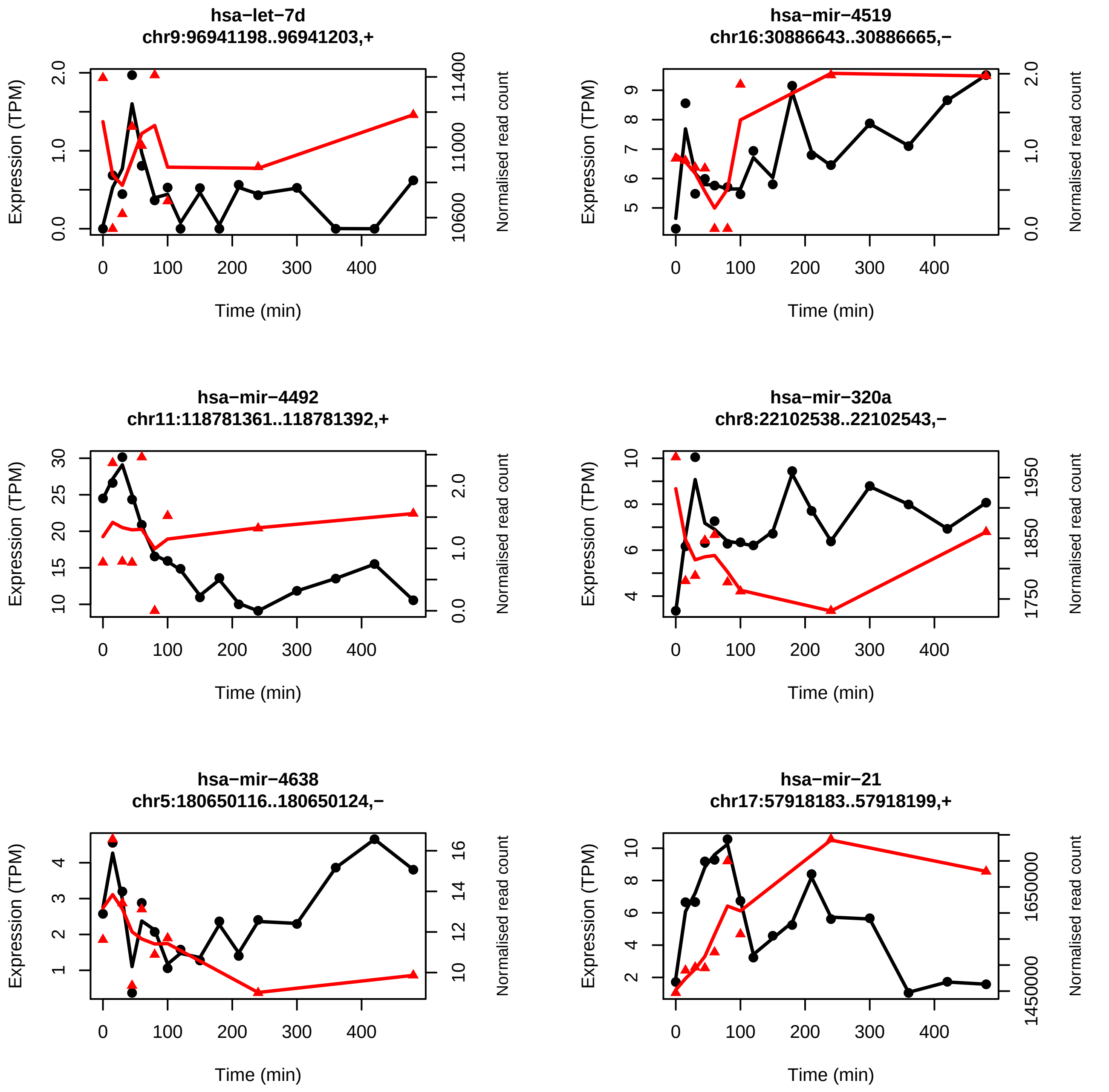

hsa−let−7d
chr9:96941198..96941203,+
hsa−mir−4519
chr16:30886643..30886665,−
hsa−mir−4492
chr11:118781361..118781392,+
hsa−mir−320a
chr8:22102538..22102543,−
hsa−mir−4638
chr5:180650116..180650124,−
hsa−mir−21
chr17:57918183..57918199,+
Expression (TPM)
Normalised read count
Time (min)

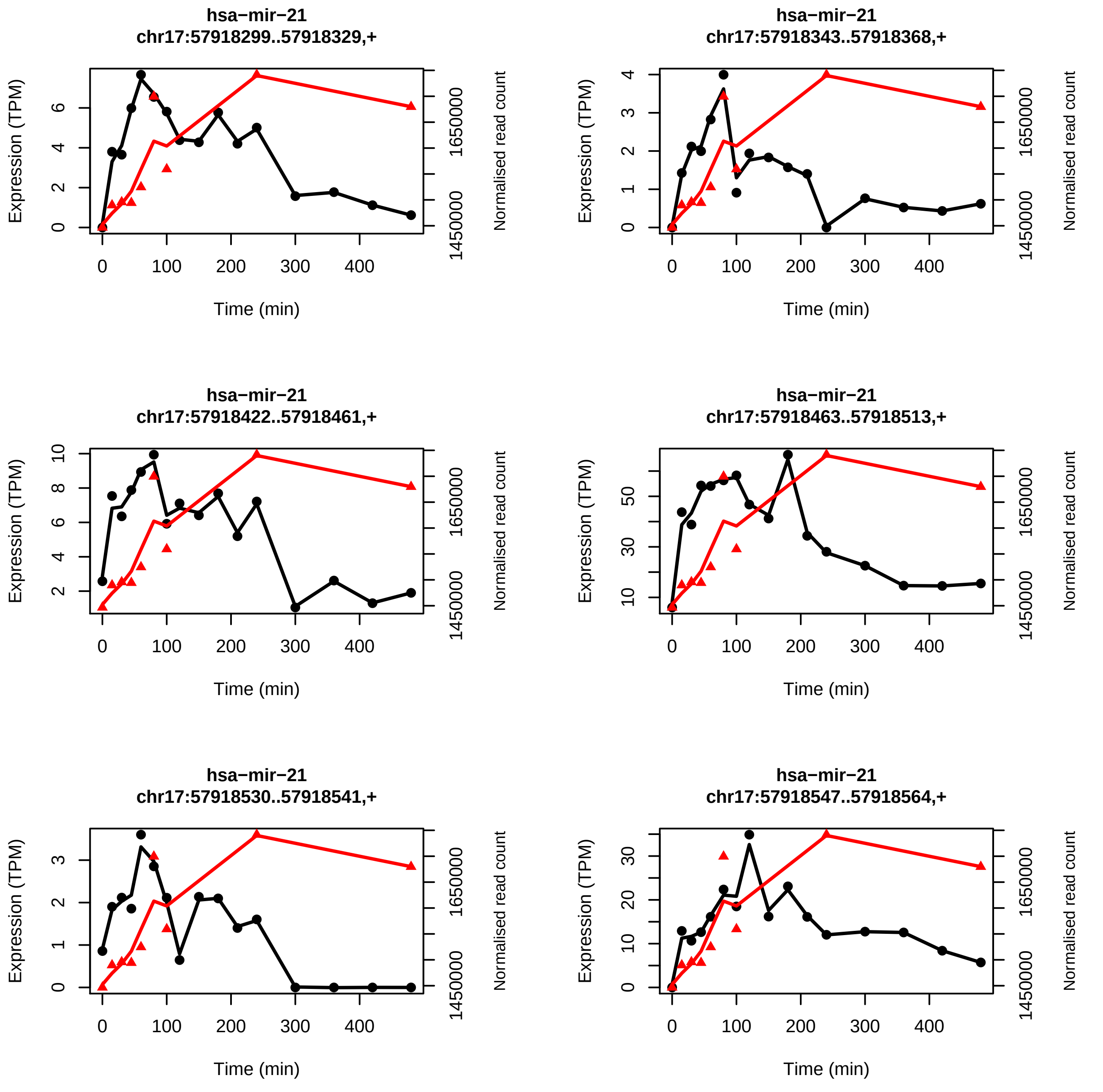

hsa−mir−21
chr17:57918299..57918329,+
Expression (TPM)
0
2
4
6
Normalised read count
1450000
1650000
0
100
200
300
400
Time (min)
hsa−mir−21
chr17:57918343..57918368,+
Expression (TPM)
0
1
2
3
4
Normalised read count
1450000
1650000
0
100
200
300
400
Time (min)
hsa−mir−21
chr17:57918422..57918461,+
Expression (TPM)
2
4
6
8
10
Normalised read count
1450000
1650000
0
100
200
300
400
Time (min)
hsa−mir−21
chr17:57918463..57918513,+
Expression (TPM)
10
30
50
Normalised read count
1450000
1650000
0
100
200
300
400
Time (min)
hsa−mir−21
chr17:57918530..57918541,+
Expression (TPM)
0
1
2
3
Normalised read count
1450000
1650000
0
100
200
300
400
Time (min)
hsa−mir−21
chr17:57918547..57918564,+
Expression (TPM)
0
10
20
30
Normalised read count
1450000
1650000
0
100
200
300
400
Time (min)

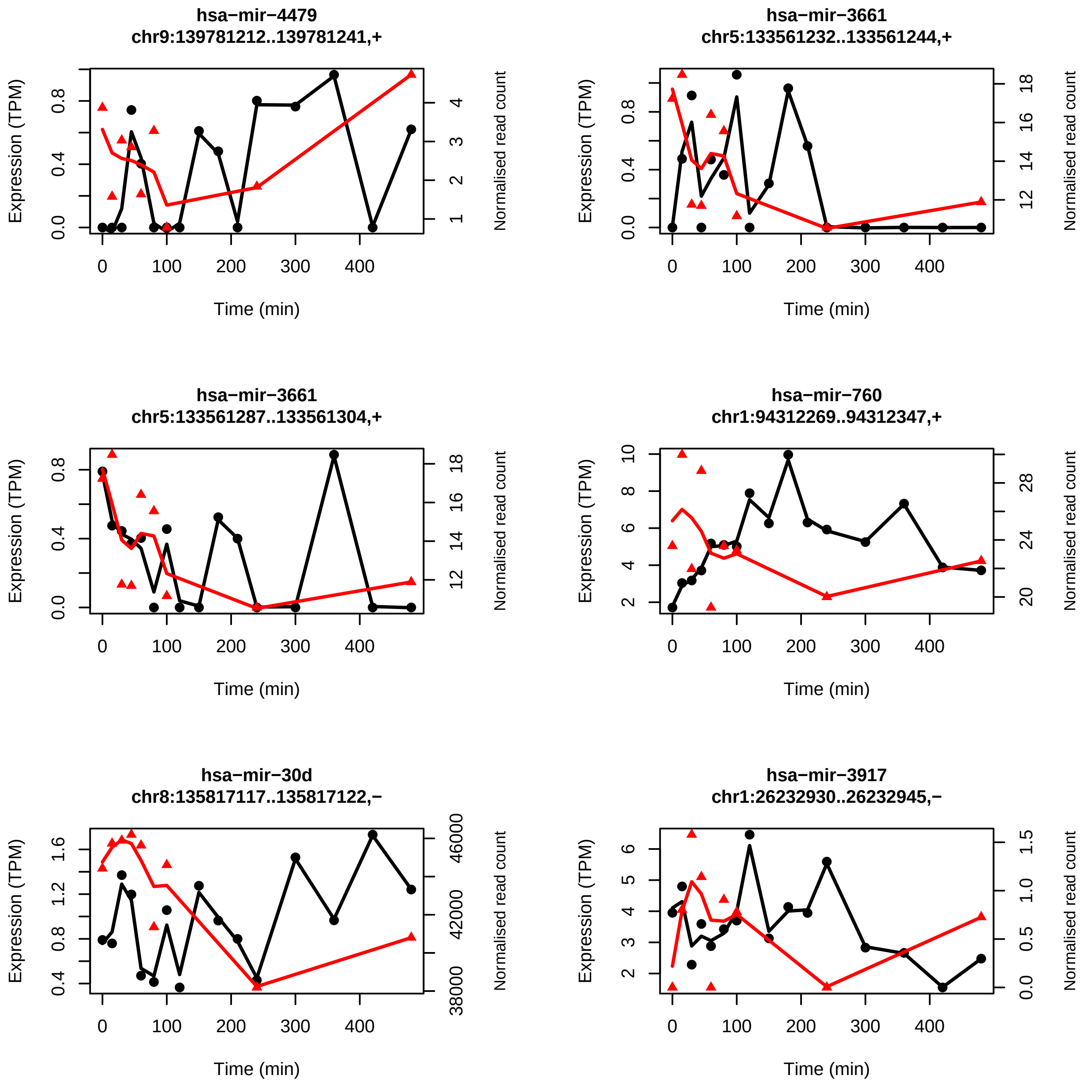
hsa−mir−4479
chr9:139781212..139781241,+
hsa−mir−3661
chr5:133561232..133561244,+
hsa−mir−3661
chr5:133561287..133561304,+
hsa−mir−760
chr1:94312269..94312347,+
hsa−mir−30d
chr8:135817117..135817122,−
hsa−mir−3917
chr1:26232930..26232945,−
Expression (TPM)
Normalised read count
Time (min)

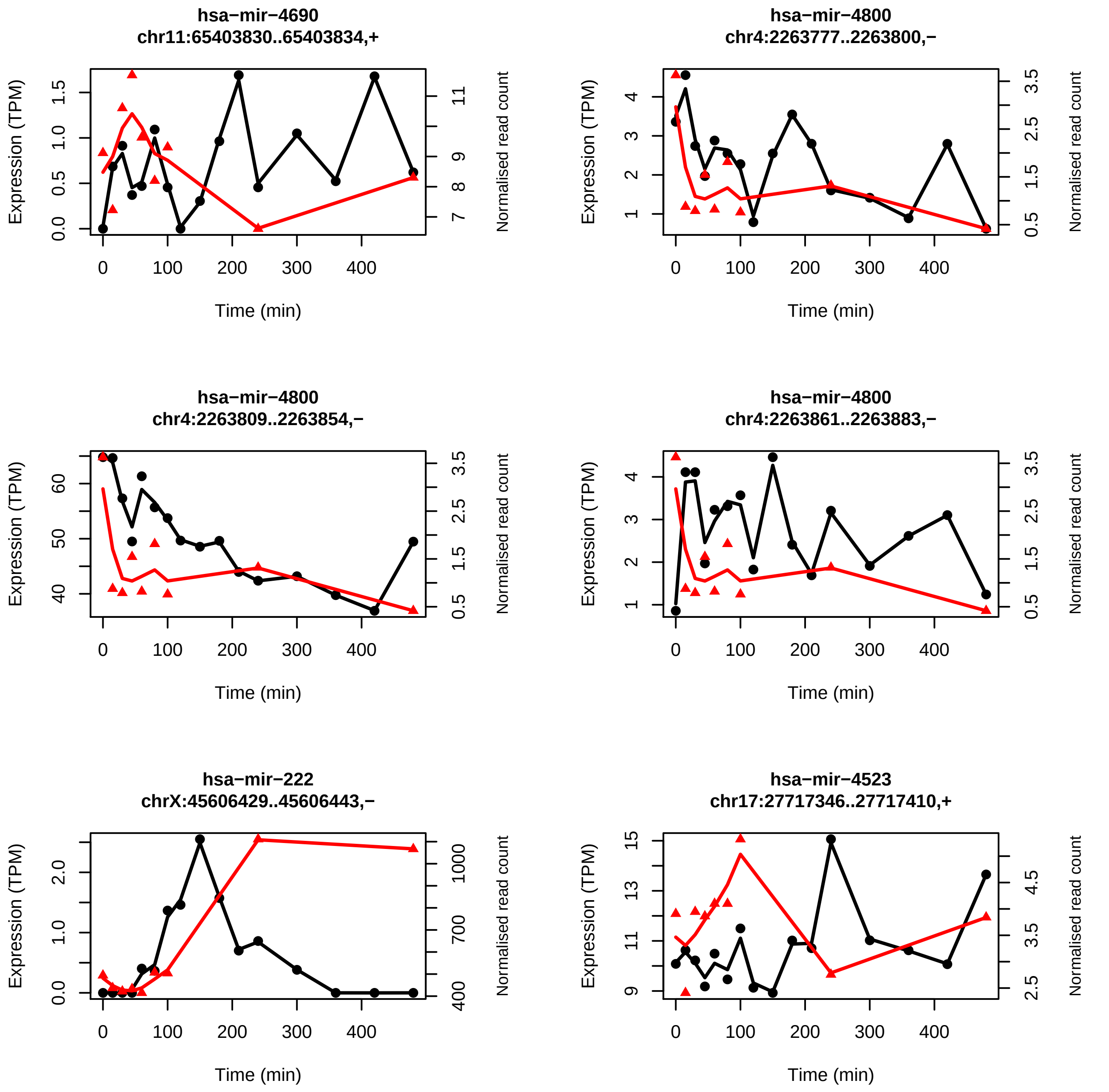
hsa−mir−4690
chr11:65403830..65403834,+
hsa−mir−4800
chr4:2263777..2263800,−
hsa−mir−4800
chr4:2263809..2263854,−
hsa−mir−4800
chr4:2263861..2263883,−
hsa−mir−222
chrX:45606429..45606443,−
hsa−mir−4523
chr17:27717346..27717410,+
Expression (TPM)
Normalised read count
Time (min)
0
100
200
300
400

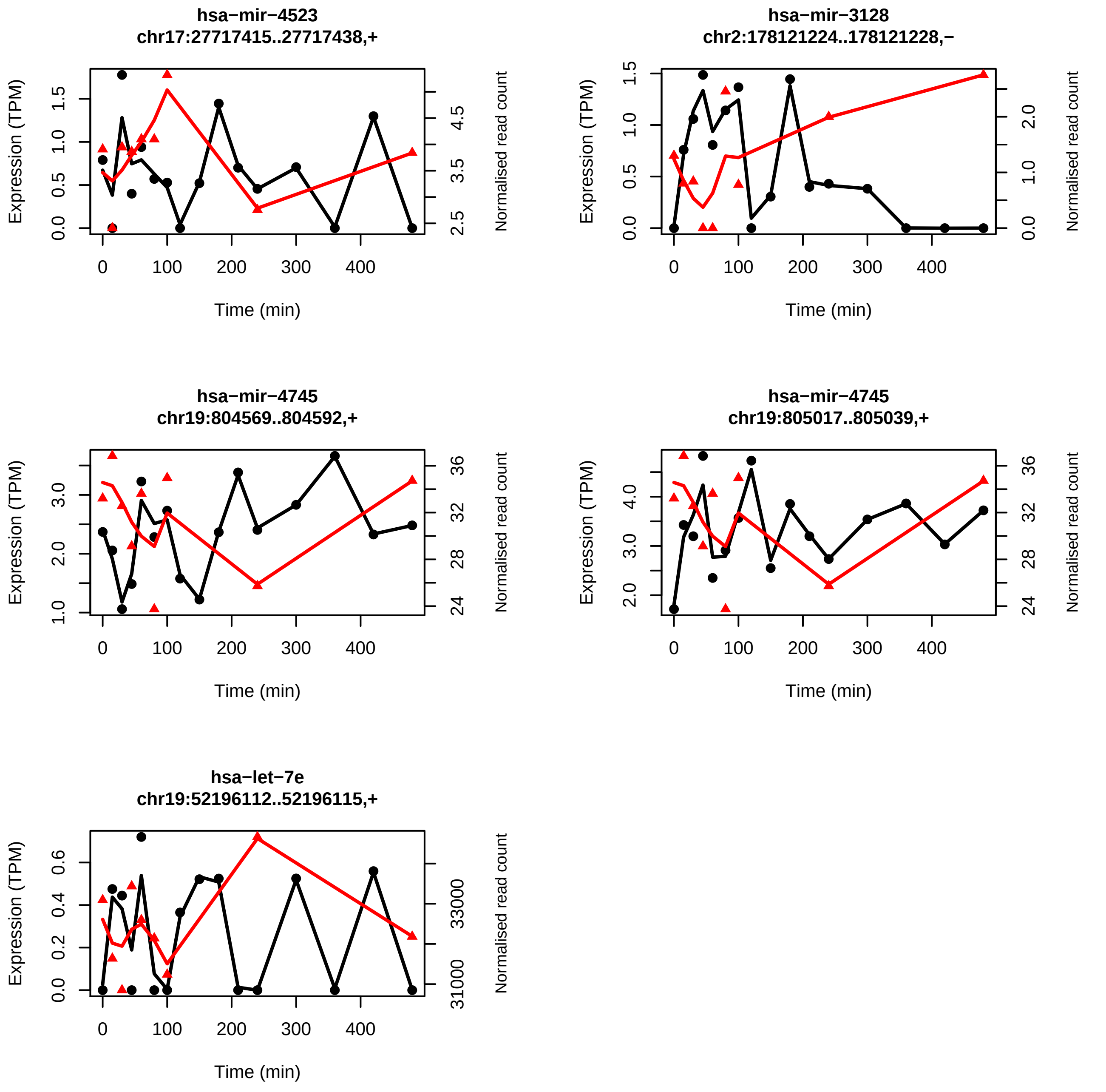
hsa−mir−4523
chr17:27717415..27717438,+
hsa−mir−3128
chr2:178121224..178121228,−
hsa−mir−4745
chr19:804569..804592,+
hsa−mir−4745
chr19:805017..805039,+
hsa−let−7e
chr19:52196112..52196115,+
Expression (TPM)
Normalised read count
Time (min)
0
100
200
300
400
