# Supplementary material for: Transcriptional Dynamics Reveal Critical Roles for Non-coding RNAs in the Immediate-Early Response
Source: PLoS Comput Biol. 2015 Apr 17;11(4):e1004217. doi: 10.1371/journal.pcbi.1004217 (PMC4401570; doi:10.1371/journal.pcbi.1004217)
Supplement: S1 Table — (PDF) [file pcbi.1004217.s017.pdf]

GO Id	Term	P value	Q value
GO:0051252	regulation of RNA metabolic process	6.03E-14	5.78E-10
GO:0006355	regulation of transcription, DNA-templated	4.73E-13	2.27E-09
GO:0060255	regulation of macromolecule metabolic process	2.04E-12	3.92E-09
GO:0080090	regulation of primary metabolic process	2.96E-12	4.73E-09
GO:0010468	regulation of gene expression	3.45E-12	4.72E-09
GO:2000112	regulation of cellular macromolecule biosynthetic process	1.31E-11	1.14E-08
GO:0019222	regulation of metabolic process	5.56E-11	4.44E-08
GO:0006357	regulation of transcription from RNA polymerase II promoter	6.90E-08	3.48E-05
GO:0090304	nucleic acid metabolic process	9.38E-08	4.29E-05
GO:0016070	RNA metabolic process	1.34E-06	3.90E-04
GO:0051254	positive regulation of RNA metabolic process	3.71E-06	8.28E-04
GO:0044260	cellular macromolecule metabolic process	1.02E-04	1.27E-02
GO:0006139	nucleobase-containing compound metabolic process	1.93E-05	3.37E-03
GO:0006725	cellular aromatic compound metabolic process	3.97E-05	6.14E-03
GO:0043170	macromolecule metabolic process	4.45E-05	6.47E-03
GO:0046483	heterocycle metabolic process	5.67E-05	7.88E-03

The terms listed also have significant p values when all four data sets are considered.
